# L-DOPA increases slow-wave sleep duration and selectively modulates memory persistence in older adults

**DOI:** 10.3389/fnbeh.2023.1096720

**Published:** 2023-04-05

**Authors:** Hanna K. Isotalus, Will J. Carr, Jonathan Blackman, George G. Averill, Oliver Radtke, James Selwood, Rachel Williams, Elizabeth Ford, Liz McCullagh, James McErlane, Cian O’Donnell, Claire Durant, Ullrich Bartsch, Matt W. Jones, Carlos Muñoz-Neira, Alfie R. Wearn, John P. Grogan, Elizabeth J. Coulthard

**Affiliations:** ^1^Clinical Neurosciences, Translational Health Sciences, Bristol Medical School, University of Bristol, Bristol, United Kingdom; ^2^Digital Health, Faculty of Engineering, University of Bristol, Bristol, United Kingdom; ^3^Southmead Hospital, North Bristol NHS Trust, Bristol, United Kingdom; ^4^Department of Neurosurgery, Heinrich-Heine-University Clinic, Düsseldorf, Germany; ^5^Production Pharmacy, Bristol Royal Infirmary, University Hospitals Bristol and Weston NHS Trust, Bristol, United Kingdom; ^6^School of Computer Science, Electrical and Electronic Engineering, and Engineering Mathematics, University of Bristol, Bristol, United Kingdom; ^7^Experimental Psychology, University of Bristol, Bristol, United Kingdom; ^8^School of Physiology, Pharmacology and Neuroscience, University of Bristol, Bristol, United Kingdom; ^9^Nuffield Department of Clinical Neurosciences, University of Oxford, Oxford, United Kingdom; ^10^School of Psychology, Trinity College Dublin, Dublin, Ireland

**Keywords:** sleep, memory, dopamine, ageing, slow wave sleep, NREM, levodopa, learning

## Abstract

**Introduction:**

Millions of people worldwide take medications such as L-DOPA that increase dopamine to treat Parkinson’s disease. Yet, we do not fully understand how L-DOPA affects sleep and memory. Our earlier research in Parkinson’s disease revealed that the timing of L-DOPA relative to sleep affects dopamine’s impact on long-term memory. Dopamine projections between the midbrain and hippocampus potentially support memory processes during slow wave sleep. In this study, we aimed to test the hypothesis that L-DOPA enhances memory consolidation by modulating NREM sleep.

**Methods:**

We conducted a double-blind, randomised, placebo-controlled crossover trial with healthy older adults (65–79 years, *n* = 35). Participants first learned a word list and were then administered long-acting L-DOPA (or placebo) before a full night of sleep. Before sleeping, a proportion of the words were re-exposed using a recognition test to strengthen memory. L-DOPA was active during sleep and the practice-recognition test, but not during initial learning.

**Results:**

The single dose of L-DOPA increased total slow-wave sleep duration by approximately 11% compared to placebo, while also increasing spindle amplitudes around slow oscillation peaks and around 1–4 Hz NREM spectral power. However, behaviourally, L-DOPA worsened memory of words presented only once compared to re-exposed words. The coupling of spindles to slow oscillation peaks correlated with these differential effects on weaker and stronger memories. To gauge whether L-DOPA affects encoding or retrieval of information in addition to consolidation, we conducted a second experiment targeting L-DOPA only to initial encoding or retrieval and found no behavioural effects.

**Discussion:**

Our results demonstrate that L-DOPA augments slow wave sleep in elderly, perhaps tuning coordinated network activity and impacting the selection of information for long-term storage. The pharmaceutical modification of slow-wave sleep and long-term memory may have clinical implications.

**Clinical trial registration:**

Eudract number: 2015-002027-26; https://doi.org/10.1186/ISRCTN90897064, ISRCTN90897064.

## Introduction

Dopamine is a neurotransmitter that plays a crucial role in various aspects of behaviour, including motor control, regulation of sleep-wake cycles (Oishi and Lazarus, 2017), learning, and memory (Lisman et al., 2011). While medications that mimic dopamine, such as L-DOPA, are commonly prescribed to individuals with Parkinson’s disease (PD) or restless legs syndrome, the effects of exogenous dopamine on human sleep and memory are not yet fully understood.

In our previous study, we showed that administering L-DOPA to individuals with PD at night results in enhanced memory, whereas taking it during the day worsened it (Grogan et al., 2015). The timing of L-DOPA administration with respect to learning and sleep critically determines its impact on memory (Rossato et al., 2009). Exogenous dopamine in humans might hinder initial learning but benefit long-term memory retention (Lisman and Grace, 2005; Feld et al., 2014; Grogan et al., 2015). Across species, dopamine modulates memory persistence *after* initial learning, possibly during replay (Frey and Morris, 1997; Chowdhury et al., 2012; Oudiette et al., 2013; Franca et al., 2015; Wimber et al., 2015; Liu and Dan, 2019; Gonzalez et al., 2021). These findings prompted the current study to understand how nocturnal L-DOPA affects memory and sleep physiology overnight.

Physiological events during non-REM sleep include delta or slow oscillations (SOs; 0.16–1.25 Hz), sleep spindles (11–17 Hz) and sharp wave ripples (80–120 Hz), all of which are associated with memory stabilisation. Memory persistence is a dynamic process that continues after learning, including during subsequent periods of sleep (Liu and Dan, 2019). As memory engrams evolve, patterns of activation within hippocampal neuronal assemblies corresponding to newly acquired memories are selectively replayed (Skaggs and McNaughton, 1996; Liu and Dan, 2019; Feld and Born, 2020), with the repetition strengthening the memory trace (Michon et al., 2019). Whilst replay occurs during both sleep and wake, SOs during sleep afford the optimal neurophysiological state for this replay to occur (Oudiette et al., 2013).

Physiologically, replay often takes place during sharp wave ripples which are, in turn, temporally coupled to sleep spindles and SOs, prominent during non-REM sleep. Thalamic dopamine is critical for the generation of SOs (Zhang et al., 2009). The increase in these delta oscillations further increases hippocampal activity during sleep, which can lead to an increase in thalamic dopamine activity, completing a self-propagating loop (Legault et al., 2000; Floresco et al., 2001; Lisman et al., 2010). Dopamine release to the hippocampus may increase long-term potentiation and learning (Lisman and Grace, 2005), particularly after learning and during sleep (O’Neill et al., 2010; Lisman et al., 2011). Therefore, dopamine or L-DOPA during sleep may increase delta activity and boost sleep-dependent memory.

In humans, retroactive retrieval of already learnt information has been shown to simultaneously enhance memory for the retrieved information while inducing forgetting for contextually related stimuli (Anderson et al., 1994). This retrieval induced forgetting is caused by an active inhibition of the competing memory traces during recall (Anderson et al., 2000; Wimber et al., 2015), and it could involve the cortical dopamine system (Wimber et al., 2011). While some evidence suggest that this memory inhibition of weaker engrams is increased by sleep (Abel and Bauml, 2012), one study found that sleep’s benefit on memory was larger for weakly, as opposed to strongly, encoded engrams in young adults (Denis et al., 2020). Therefore, it needs to be tested whether L-DOPA administration will enhance sleep systems that promote retention of weakly encoded engram, or whether dopamine biases memory toward more strongly encoded information.

Here, we test how administering L-DOPA to increase dopamine levels in older humans affects sleep and memory. Our specific hypothesis is that increasing dopamine levels after learning by administration of L-DOPA will enhance recognition of stronger (re-exposed) memories at the expense of weaker memory traces and that this will depend on dopaminergic alteration of slow-wave activity and associated spindles during sleep.

## Materials and methods

To study the relationships between dopamine, sleep and memory, we carefully timed administration of L-DOPA to increase dopamine concentration in healthy older adults across two placebo-controlled double-blind randomised crossover experiments.

**FIGURE 1:**
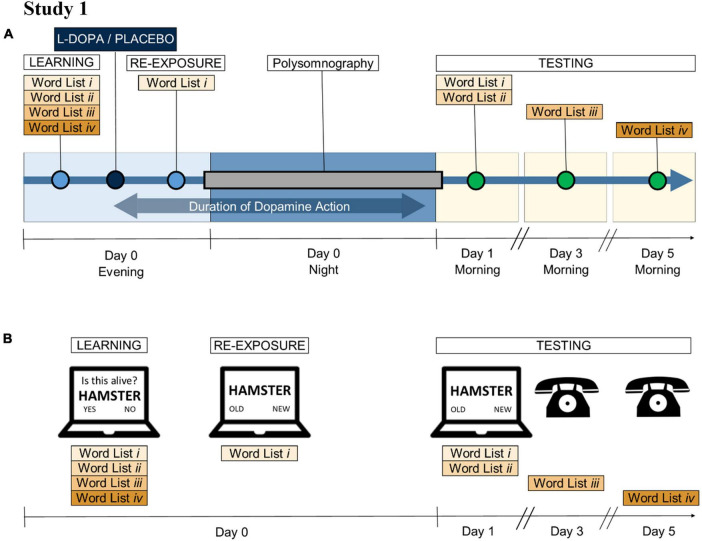
Main study procedure. **(A)** Study 1 schedule: In the evenings, participants learnt a set of words (Lists *i* to *iv*) 45 min *before* receiving 200 mg L-DOPA CR or placebo. Seventy-five minutes *after* dosing a proportion of the words were re-exposed. Their memory on the items was tested 1 (Lists *i* and *ii*), 3 (List *iii*), and 5 (List *iv*) days later. The blue double-headed arrow denotes when L-DOPA was active (during re-exposure and sleep but not during learning or memory tests). The same group of participants completed both the L-DOPA and the placebo conditions, following the same schedule. There was a minimum 7-day washout period between the sessions. **(B)** Wordlist memory task: Participants were asked to memorise 80 words, which were presented in random, interleaved order but separated into four lists for testing (Lists *i*, *ii*, *iii*, *iv*—20 words each). Two hours after learning, participants were re-exposed to List *i* by a recognition test. The following morning, memory for Lists *i* and *ii* was tested (random, interleaved), while lists *iii* and *iv* were tested 3 and 5 days later over the phone. Each recognition test was performed with a unique set of distractor words, matched to the number of target words.

In the main experiment (Study 1: Figure 1A), we investigated the effects of L-DOPA on sleep and memory consolidation by administering long-acting L-DOPA to be active after initial learning and during nocturnal sleep. In a second experiment, we used short-acting L-DOPA to target memory retrieval (testing) or encoding (learning), but not sleep (Hsu et al., 2015). Full method is given in Supplementary material 1.

### Participants

We recruited 70 elderly (65 + years) participants to complete the two studies (*n* = 35 each study, Supplementary materials 2, 3). We sampled healthy elderly because older people have relative age-related dopamine depletion. Therefore, dopamine replacement therapies are less likely to “overdose” the dopamine system, which could mask effects (Vijayraghavan et al., 2007; Cools and D’Esposito, 2011; Chowdhury et al., 2012). Sample sizes were determined by a power calculation based on previous work (Grogan et al., 2015), suggesting a minimum sample size of 25 (μ (0) = 53.2, μ (1) = 62.4, σ = 18.6) in detecting L-DOPA’s effects on verbal memory using the conventional threshold for power (α = 0.05, β = 0.80) in a similar memory task to the one reported here. Both studies were placebo-controlled, crossover, double-blind and order randomised. Each participant therefore received both placebo and L-DOPA but on different study visits.

All aspects of this research adhered with the Declaration of Helsinki and relevant ethical and regulatory (UK) approvals were in place. Study 1 formed a part of a clinical trial registered with ISRCTN (ISRCTN90897064) and EudraCT (2015-002027-26).

### Procedure and materials

#### Study 1 (main experiment)

Participants were invited for three in-house visits: a screening visit, and two overnight in-laboratory sleep study visits at the Clinical Research and Imaging Centre (CRIC) in Bristol (Figure 1A). On the evenings of the sleep visits, participants were presented with four word lists (Lists *i* to *iv*) as part of a verbal memory task (Figure 1B and Supplementary material 4). Thirty minutes later they were given 200 mg long-acting L-DOPA (CR; co-beneldopa 200/50 mg) or placebo. The timing of dosing was designed to test impact of L-DOPA after initial learning. After a 75 min interval following dosing, a quarter of the items (List *i*) were re-exposed by a practice test where no feedback was given. The purpose of this test was to create a stronger memory trace for re-exposed words. After a 45 min interval following re-exposure the participants went to bed, at their usual time. Participants then slept on-site for a full night, and they were woken up at their usual wake-up time. Participants’ verbal memory was tested again in the morning (Lists *i* and *ii*), and 3 and 5 days after dosing and learning (Lists *iii* and *iv*, respectively by telephone). Participants were invited for a second sleep visit which was identical except for L-DOPA/placebo allocation.

Note that initial learning occurred *before* L-DOPA (or placebo) administration, whereas memory re-exposure and a full night of sleep occurred *after* L-DOPA (or placebo) administration. Items presented only once (Lists *ii–iv*) were expected to have induced weaker memory traces than the re-exposed items (List *i*). Testing over several days (Days 1, 3, and 5) allowed us to measure memory decay.

Polysomnography, including video, was recorded during both study nights using the Embla N9000 amplifier and Embla RemLogic software (Natus Medical Inc., CA, USA) at CRIC Bristol, University of Bristol, Bristol, UK. We recorded 12 scalp EEG channels (F3, Fz, F4, C3, Cz, C4, M1, Pz, M2, O1, O2, and a ground electrode approximately between Cz/P3 and C3/Pz) placed according to the 10-20 system with eye movements (E1 and E2), chin EMG and 2-lead ECG. All signals were sampled at 500 Hz. The recordings started at the lights out time, which was 2.5h after dosing, and continued through until morning wake-up time.

Participants also completed a visual reward memory task. However, the reward manipulation was unsuccessful and therefore it was not analysed further (*SM5*).

#### Study 2 (control experiment)

Participants completed two test sets. Each set carried over for three days and followed the same format (Supplementary material 6).

On Day-1 (Day “minus one”; relative to dosing), following a screening procedure for eligibility, participants learnt a word list on-site before returning home. On Day 0 they returned on site where they were dosed with 150 mg L-DOPA (in form of co-beneldopa) or placebo. They were then tested on the previously learnt words. The purpose of this test was to examine L-DOPA’s effects on retrieval.

Shortly following the test, and whilst L-DOPA was still active, they learnt another word list. On Day 1, participants’ memory was tested over the phone for the words learnt on the previous day. The purpose of this test was to examine L-DOPA ‘s effects on encoding.

Therefore, participants learnt two word lists on each test with 24 h in between learning and test. For one of the tests participants were dosed before testing to target retrieval, for the other they were dosed before learning to target encoding. The second test set was identical to the first one except this time participants received placebo (or 150 mg co-beneldopa; order randomised and double-blinded).

Note that the two studies had a different doses and preparations. In Study 1, participants received long-acting formulation with an elimination half-life of ∼5 h, while in Study 2 participants received standard formulation with a half-life of ∼1.5 h. This was in order to delineate acute effects of L-DOPA during retrieval rather than prolonged effects during consolidation and sleep.

### Analysis

We used d’ (D-prime) as a measure of recognition memory accuracy in both studies. D’ is a sensitivity index that takes into account both the accurately detected signal (hits) and inaccurately identified noise (false alarms) (Birdsall and Roberts, 1965). Higher d’ indicates better accuracy at performing the task, while 0 indicates performance at chance.

Sleep stages in Study 1 were scored manually by expert scorers. Durations of N1, N2, N3 (i.e., slow-wave sleep), REM, wake, total sleep time and time in bed were extracted in minutes. First and second halves of the nights were defined by the middle time-point between switching lights ON and OFF.

Spindle and SO events were automatically detected using similar criteria to comparable studies (Phillips et al., 2012; Helfrich et al., 2018) with in-house developed software, from the Cz electrode location (Supplementary material 1). We identified spindle/SO co-occurrences as cases where the maximum amplitude of a spindle event coincided with a SO event. Using the time stamp of the spindle max amplitude as the centre point, we calculated how spindle amplitude varied with SO phase over one cycle. First, we divided the oscillation events into 16 bins, equally distributed in phase space around zero, to calculate how the spindle amplitude varied with SO phase for each coinciding case. Then, for statistical analyses we grouped together 4 adjacent frequency data points (bins) to generate a total of 4 bins.

The spectral composition of each spindle-SO co-occurrence was done using a Morlet wavelet time-frequency method over a 4s window centred at the SO peak. A spectral mean was then calculated for each participant for L-DOPA and placebo conditions separately. We identified power differences using a cluster-based permutation method (Oostenveld et al., 2011). Based on the finding that maximum spindle amplitude occurring near the SO peak predicts memory performance in ageing (Helfrich et al., 2018), an *a priori* spindle region of interest of 11–16Hz, −0.5 to 0.5 s was chosen for initial analyses. This same cluster method was then carried out on the wider time-frequency space, 1–20Hz, −1 to 1 s, the primary cluster (*p* = 0.002, see results below).

As part of an exploratory analysis, spectral power in the 0.6–1 Hz and 1–4 Hz bands during N3 sleep were calculated to allow for direct comparison with previously published methodology (Mander et al., 2015). We used the Hamming Method with a 500 ms window size and 250 ms overlap, returning power in 0.1 Hz increments.

False discovery rate adjustment was performed for dependent tests using the Benjamini–Hochberg procedure (Benjamini and Hochberg, 1995). Where *p*-values have been corrected using this method, they are reported alongside the number of dependent tests.

## Results

Thirty-five adults completed the main study (Study 1, age = 68.9 ± 3.5 years; 22 Female, Supplementary material 3).

### L-DOPA worsens some aspects of memory during sleep

**FIGURE 2:**
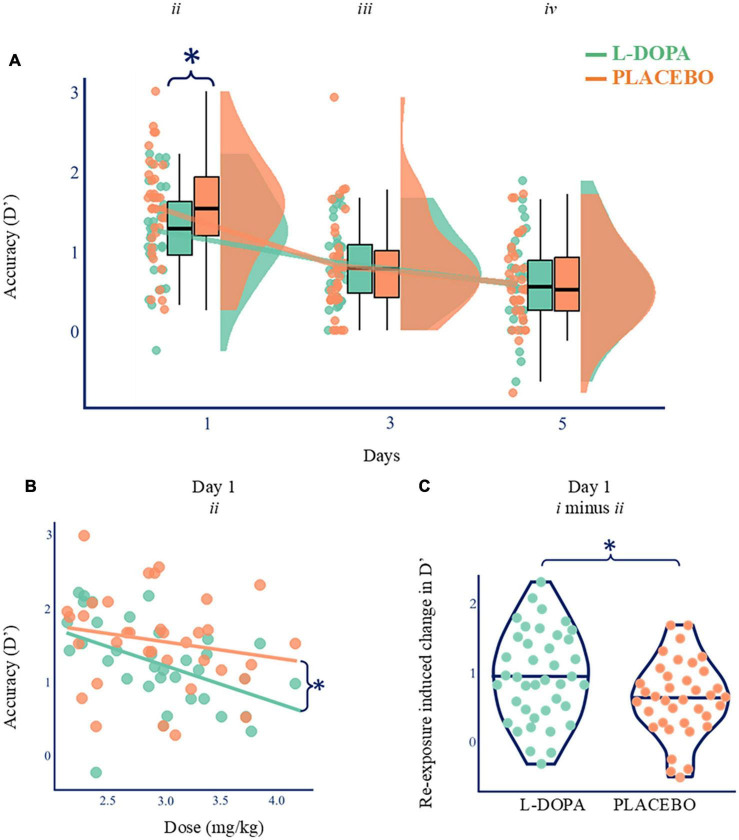
Nocturnal dopamine dose-dependently modulates memory. **(A)** Paired comparisons for d’ across time. D’ was higher on Day 1 on placebo (orange; d’ mean ± standard deviation = 1.54, ±0.65) compared with L-DOPA (green; mean = 1.25 ± 0.59; Wilcoxon’ = 121, *p* = 0.004, BF_10_ = 16.6, Cohen’s δ = 0.46, *n* = 35), but there was no difference on Days 3 or 5 (*p* > 0.05). Boxplots show quartiles with kernel densities plotted to the right. *p < 0.05. **(B)** Higher L-DOPA dose during consolidation correlated with poorer Day 1 recall of single exposure items (List *ii* d’; Spearman’s ρ = –0.560, *p* < 0.001, *n* = 35) but no such relationship was found on the placebo night (ρ = –0.231, *p* = 0.180, orange). These two relationships were also different [r-to-z transform z = –2.554, *p* = 0.011, (Lee and Preacher, 2013)]. Lines of best fit presented for illustration purposes. *p < 0.05. **(C)** L-DOPA increased the relative benefit of re-exposed compared to other items (List *i* d’ minus List *ii* d’; Wilcoxon = 444, *p* = 0.034, BFio = 2.6, Cohen’s δ = 0.42, *n* = 35, SM9). Lines show maximum, median, and minimum values (horizontally) and kernel densities (vertically). *p < 0.05.

First, to study the effect of L-DOPA on consolidation, we examined next day memory for the words that had been learnt before dosing. These analyses only included words that had not been repeated in the evening (List *ii*). D’ for remembered words was reduced following L-DOPA (**d’**_*List*_ *_*ii*_* = 1.25 ± 0.59, mean ± standard deviation) compared to placebo (**d’**_*List*_ *_*ii*_* = 1.54, ± 0.65) at Day 1 (Wilcoxon test statistic (W) = 121, *p* = 0.004, p_*corrected*_ = 0.012, n_*dependent tests*_ = 3, BF_10_ = 16.6, Cohen’s δ = 0.46). By Day 3 there was no difference (**d’**_*List*_ *_*iii*_*: L-DOPA = 0.86 ± 0.46; placebo = 0.82 ± 0.63; W = 338, *p* = 0.313, BF_01_ = 5.2, *n* = 35; Day 5: **d’**_*List*_ *_*iv*_*: L-DOPA = 0.58 ± 0.58; placebo = 0.59 ± 0.55; W = 273, *p* = 0.682, BF_01_ = 5.4). These findings show that L-DOPA accelerates the initial speed of forgetting over 1 night, but this information would be lost in the longer term even without L-DOPA (Figure 2A and Supplementary materials 7, 8). Paired comparisons, as opposed to ANOVAs, were used due to missing data (*SM9*) for a single Day 5 (List *iv*) memory test.

Whilst every participant was administered the same dose of L-DOPA, differences in participant weight were predicted to have pharmacodynamic and pharmacokinetic effects on effective dose. Previously, exogenous dopamine’s effects on cognition have been reported to be dose-dependent in animals (Vijayraghavan et al., 2007) and in humans (Cools and D’Esposito, 2011; Chowdhury et al., 2012). Mixed linear effects allow including participant-level information, such as drug dose, in the model. Therefore, a mixed linear model with weight-adjusted dose (mg/kg), delay from learning (number of days) and the interaction term (delay × dose) as fixed effects and participants as random effects (including random intercepts and slopes of delay by participant) revealed a main effect of delay [*t*(33.7) = −9.142, *p* < 0.001], no overall effect of dose [*t*(20.3) = −1.36, *p* = 0.188] and a delay × dose interaction [*t(*98.2) = 2.33, *p* = 0.022, *n* = 35, Table 1].

**TABLE 1:** L-DOPA accelerates memory decay.

A	*Accuracy* ∼ Delay * Dose + (*Delay* + *Dose | | participant*)					
	**Estimate (std error)**	***t***	**df**	***p***	***p*** **(corrected)**	***R*** **^2^ marginal**
**Forgetting**						
Intercept	0.940 (0.06)	14.931	34.0	< 0.001	<0.001	0.258
Delay (days)	−0.808 (0.09)	−9.142	33.7	<0.001	<0.001	
Dose	−0.031 (0.02)	−1.364	20.3	0.188	0.188	
Delay × dose	0.123 (0.05)	2.325	98.2	0.022	0.029	
**B**	**Mean (SD)**		**Credible interval δ**	**df**	***t***	***p***	***p*** **(corrected)**	**BF_01_**
	**L-DOPA**	**Placebo**						
Day 1	1.249 (0.59)	1.544 (0.65)	[−1.202 to −0.232]	34	−3.333	0.002	0.006	<0.1**
Day 3	0.855 (0.46)	0.818 (0.63))	[−0.360 to 0.508]	34	337.5*	0.313	0.470	5.2
Day 5	0.584 (0.58)	0.593 (0.55)	[−0.434 to 0.428]	33	−0.023	0.982	0.982	5.4

A. A mixed linear model was used to observe the impact of dose on memory performance over time. We included delay (days 1, 3, or 5), dose (mg/kg) and delay × dose interaction as fixed effects, with participants as random effects (including slopes and intercepts). Delay and delay * dose interaction explained variability in accuracy, but with no main effect of dose. R^2^m quantifies extent of variance in accuracy explained by the fixed effects, their interactions and the intercept. Note that estimates are mean-centred. Top line of table A denotes model specification in R. *p*-Values are FDR corrected (n tests = 4) for the whole model using Benjamini-Hochberg procedure. B. These *post-hoc* exploratory comparisons show that L-DOPA compared to placebo administered after learning lowered d’ on day 1 but not at later times. *p*-Values are FDR corrected for days (n tests = 3) using Benjamini–Hochberg procedure. Δ denotes effect size for the paired differences derived from the Bayesian posterior distribution. Credible intervals overlapping zero denote no difference. All errors < 0.001%. Note that BF_01_ which denotes how much likelier our data are under the null are reported as opposed to BF_10_ for easier interpretation. An unplanned additional ANOVA was performed. It supported our findings and revealed a main effect of time [*F*(2, 66) = 40.31; *p* < 0.001] and a time × treatment condition interaction [*F*(2, 66) = 3.15; *p* = 0.049] but no main effect of treatment [*F*(1,33) = 1.51; *p* = 0.228]. *Wilcoxon test used. The sample contained zero values for paired differences. *p*-Values for Wilcoxon tests for such data are less reliable. **BF_10_ = 16.6 (our data is 16.6 times more likely to have been drawn from the alternative than the null distribution).

Next, we explored what was driving this interaction. The degree of forgetting (**d’** on Day 1) correlated with L-DOPA dose following L-DOPA (Spearman’s ρ = −0.560, p_*corrected*_ < 0.001, n_*dependent tests*_ = 6, *n* = 35) but not following placebo (Figure 2B—Spearman’s ρ = −0.231, *p* = 0.18, *n* = 35). These two relationships were also different [r-to-z transform z = −2.554, *p* = 0.011 (Lee and Preacher, 2013)]. Therefore, this effect is not explained by body weight alone. As a robustness check, we ran a version of these analyses where we excluded datapoints that appeared as outliers. This check produced similar results. There were no correlations between **d’** and dose on days 3 or 5 in either condition (ps > 0.36). Therefore, the delay × dose interaction was driven by L-DOPA having a dose-dependent effect on memory for List *ii* on Day 1 but not at subsequent delays (in keeping with day 1 accelerated forgetting due to L-DOPA).

### L-DOPA accelerates forgetting weak but not strong engrams

Next, we examined whether L-DOPA has a disparate effect on memory persistence depending on memory strength by comparing Day 1 recognition memory for List *i* (re-exposed, i.e., shown twice) and List *ii* (shown once).

As expected, strong memories (List *i*) were better retained than weak ones (List *ii*) following L-DOPA (**d’**_*List*_ *_*i*_* = 2.20 ± 0.78; **d’**_*List*_ *_ii_* = 1.25 ± 0.59; t(34) = 8.49, p_*corrected*_ < 0.001, n_*dependent tests*_ = 4, BF_10_ = 1 430,000, Cohen’s δ = 1.42) and placebo (**d’**_*List*_ *_*i*_* = 2.19 ± 0.76; **d’**_*List*_ *_*ii*_* = 1.54 ± 0.65; t(34) = 6.76, p_*corrected*_ < 0.001, n_*dependent tests*_ = 4, BF_10_ = 166 000, Cohen’s δ = 1.14). While L-DOPA reduced recognition performance for weaker items, as shown above, re-exposed List *i* items were unaffected (**d’**_List_*_i_* = 2.20 ± 0.78; placebo **d’**_*List*_ *_i_* = 2.19 ± 0.77; t(34) = 0.134, *p* = 0.894, BF_10_ = 5.5). Further, the difference in performance between stronger and weaker engrams (i.e., d’ for List *i* minus d’ for List *ii*) was higher on L-DOPA (**d’**_*List*_ *_*i*_*_*minus List*_ *_*ii*_* = 0.95 ± 0.67) than on placebo (**d’**_*List*_ *_*i*_*_*minus List*_ *_*ii*_* = 0.64 ± 0.56; W = 444, p = 0.034, BF_10_ = 2.6, Cohen’s δ = 0.42, *n* = 35, Figure 2C and Supplementary materials 10–12). As we had considered retrieval-induced forgetting might, to some extent, rely on dopamine, we expected L-DOPA to enhance retention for strong engrams while leaving weaker memories unaffected. Instead, L-DOPA accelerated memory loss for weaker information leaving stronger memories unaffected.

Control analyses showed that there was no effect of L-DOPA on **d’** during the evening re-exposure tests (Supplementary material 11). Therefore, L-DOPA did not affect memory performance before sleep − effects here only manifested after or during the re-exposure. Second, L-DOPA had no effect on false alarm rate, but reduced the hit rate compared to placebo (false alarms: t(34) = 0.527, *p* = 0.601, BF_01_ = 4.8; hits: List *ii* – t(34) = −2.89, *p* = 0.007 p_*corrected*_ = 0.014, n_*dependent tests*_ = 2, BF_10_ = 6.0, Cohen’s δ = 0.488). This shows that the effect was dependent on memory persistence as opposed to a more liberal response strategy during recognition test.

### L-DOPA prolongs slow-wave sleep

**FIGURE 3:**
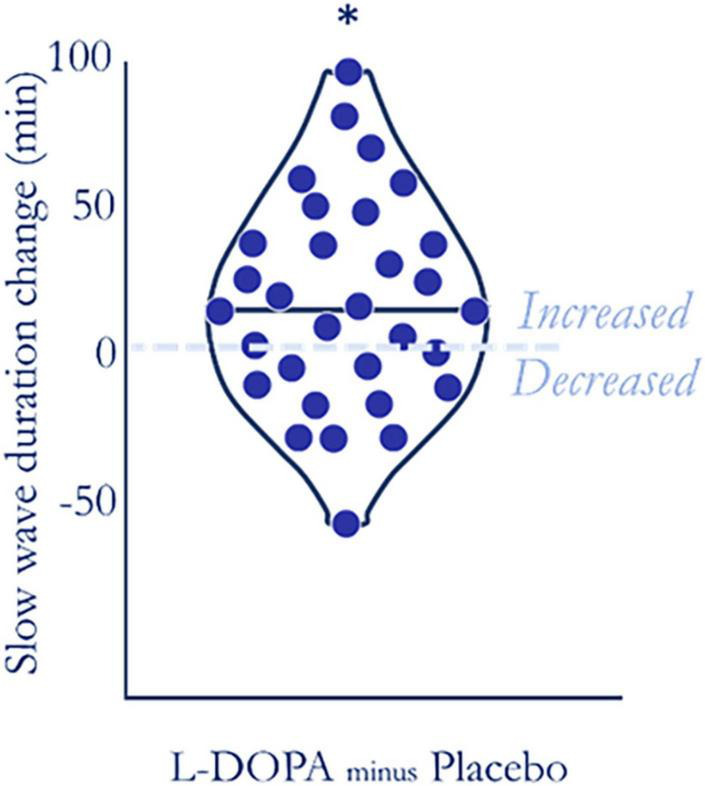
L-DOPA increased slow-wave sleep duration. The majority of participants (20 of 31—dots above zero) had increased slow-wave sleep duration on L-DOPA compared to placebo. Duration increased by an average of ∼10.6% (t(31) = 2.702, *p* = 0.011, BF10 = 4.0, SM8), even after false discovery rate collection accounting for each sleep stage (p_corrected_ = 0.033).

The polysomnography recordings revealed that nocturnal L-DOPA increased slow-wave sleep (N3) duration by ∼10.6% (Figure 3) but it did not markedly affect durations of other sleep stages (Table 2). This L-DOPA induced increase in slow wave sleep duration was not correlated with weight-adjusted dose (Spearman’s ρ = −0.048, *p* = 0.796, *n* = 31). As most slow wave sleep occurs in the first 4 h of sleep and the absorption profile of L-DOPA controlled release strongly predicts that dopamine would be increased in the first half of the night (Hsu et al., 2015), we expected that L-DOPA would predominantly affect sleep during this time. Consistent with L-DOPAs half-life profile, we observed an increase in slow-wave sleep only during the first half of the night on L-DOPA (90.2 ± 34.1 min) compared to placebo [76.8 ± 30.3 min, (t(30) = −3.07, *p* = 0.005, p_*corrected*_ = 0.033, n_*dependent tests*_ = 3, BF_10_ = 8.7, n = 31, Cohen’s δ = 0.48, for missing data see Supplementary material 9). L-DOPA had no effect on slow-wave sleep duration during the second half [t(30) = −0.387, *p* = 0.703, BF_01_ = 4.9), when there would have been less L-DOPA active in the system.

**TABLE 2:** Single dose of nocturnal L-DOPA increases time spent in slow wave sleep by 10.6%.

Time in minutes	Mean (SD)		Credible interval δ	t	*p* (uncorrected)	BF_01_
	**L-DOPA**	**Placebo**		**df = 31**		
Total time in bed time	581.1 (200.3)	522.9 (144.2)	[−0.269 to 0.650]	365.5*	0.067	3.6
Asleep	363.7 (60.4)	358.0 (53.8)	[−0.368 to 0.543]	0.402	0.691	4.9
Awake After Sleep Onset	116.6 (46.6)	110.5 (50.9)	[−0.272 to 0.636]	0.838	0.408	3.8
N1	20.0 (8.4)	21.9 (8.8)	[−0.712 to 0.210]	−1.160	0.255	2.9
N2	140.9 (51.1)	140.6 (58.2)	[−0.574 to 0.327]	−0.576	0.569	4.5
N3 (SWS)	132.8 (54.0)	120.0 (51.3)	[0.116 to 1.104]	2.702	0.011**	0.2***
REM	70.0 (24.7)	75.5 (25.1)	[−0.768 to 0.142]	−1.426	0.164	2.1

L-DOPA increased time spent in slow wave sleep, but it did not affect light sleep (stages 1 and 2), wakefulness or total time spent asleep. Δ denotes effect size for the paired differences derived from the Bayesian posterior distribution. Where credible intervals are the 95% intervals overlapping zero denote no difference. *p*-Values are uncorrected. Note that BF_01_ which denotes how much likelier our data are under the null are reported as opposed to BF_10_ for easier interpretation.

*Wilcoxon test used.

**A Benjamini–Hochberg corrected *p*-value = 0.044, when including all stages (N1, N2, N3, and REM; *n* tests = 4).

***Corresponding BF_10_ = 4.0.

All errors < 0.055%.

Exploratory analyses revealed no differences between L-DOPA and placebo on self-report sleep questionnaires (Supplementary material 13).

### L-DOPA increases 1-4Hz power during slow-wave sleep

**FIGURE 4:**
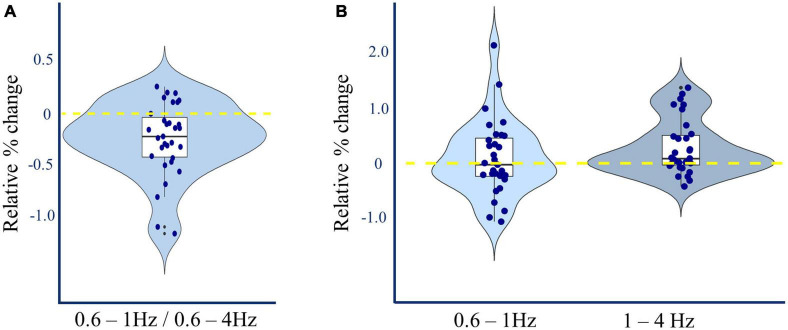
L-DOPA and non-REM spectral power. **(A)** Relative change in 0.6–1Hz/0.6–4Hz N3 Slow Wave Activity. Dopamine induced a 0.248% average reduction in the proportion of 0.6–1.0 Hz compared to 0.6–4.0 Hz spectral power in slow wave sleep averaged across all channels [paired t(30) = –3.928, Cohen’s δ = –0.22, *p* < 0.001, p_corrected_ = 0.001, n_dependent tests_ = 3]. This reduction was manifest in 24 out of 31 participants. **(B)** Relative change in N3 Slow Wave Activity by frequency. Dopamine induced a 0.30 dB mean increase in 1–4 Hz spectral power during slow wave sleep, averaged across all channels [*t*(30) = 3.206, Cohen’s δ = 0.22, *p* = 0.003, p_corrected_ = 0.005, n_dependent tests_ = 3], without corresponding increase in 0.6–1 Hz spectral power.

*Post-hoc* analysis of 0.6–1 Hz vs. 1–4 Hz spectral power during slow-wave sleep was performed to take account of previously reported findings where oscillations in these frequency bands were differentially linked to future Alzheimer’s pathology (Mander et al., 2015). Slower frequencies were not affected by L-DOPA administration (0.6–1 Hz dB L-DOPA = 25.24 ± 0.21; placebo = 25.20 ± 0.25, (t(30) = 0.341, *p* = 0.735, BF_01_ = 4.9). However, there was an increase in faster frequencies (1–4 Hz dB L-DOPA = 16.89 ± 0.22; placebo = 16.60 ± 0.23, (t(30) = 3.206, p = 0.003, p_*corrected*_ = 0.005, n_*dependent tests*_ = 3, BF_10_ = 11.9, Cohen’s δ = 0.22). These effects together reduced the proportion of 0.6–1 Hz vs. 0.6–4 Hz activity (Δ% 0.6–1 Hz proportion L-DOPA = −0.248 (t(30) = −3.928, *p* < 0.001, p_*corrected*_ = 0.001, n_*dependent tests*_ = 3, BF_10_ = 64.8, Cohen’s δ = −0.22; Figures 4A, B).

### L-DOPA increases spindle amplitude during slow-wave sleep

**FIGURE 5:**
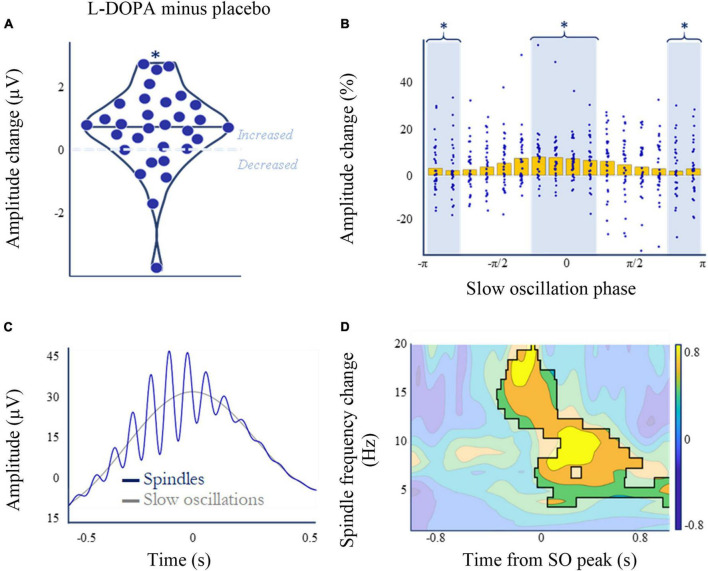
L-DOPA, memory, and spindle amplitude during slow wave sleep (N3). **(A)** Nocturnal L-DOPA increased mean spindle amplitude during slow wave sleep (Wilcoxon’s z = 401, *p* = 0.002, *p*_corrected_ = 0.008, *n*_dependent tests_ = 4, BF10 = 3.6; rank biserial correlation = 0.62). **(B)** The dopamine-induced spindle amplitude increase **(A)** was slow-wave phase-dependent. Mean spindle amplitude change (normalised to baseline amplitude ([placebo + L-DOPA]/2) is higer on L-DOPA around the zero phase of slow-waves [*t*(30) = 2.12, *p* = 0.043, BF10 = 1.3]. **(C)** Spindle amplitude peaked in the –π/4 to –π/8 phase bin for both placebo and L-DOPA. Peak locked grand average mean slow-wave events (grey) superimposed with the peak-locked average of all spindle events (blue) that occurred during slow oscillations—averaged across both L-DOPA and placebo nights. **(D)** Paired permutation cluster analysis in time-frequency space comparing L-DOPA and placebo conditions for all slow-wave - spindle co-occurrence events, centred on the slow-wave peak. All shown differences denote increased activity on L-DOPA (cluster threshold of α = 0.01, time frequency space outside significant clusters is greyed). Overall *p* = 0.002 for the largest cluster. *p < 0.05.

Spindles are a prominent feature of non-REM sleep and are associated with memory consolidation (Antony et al., 2018; Cairney et al., 2018). L-DOPA increased spindle amplitude during slow-wave sleep (N3), and while this increase was small on average (m_*placebo*_ = 28.3 ± 8.5 μV; m_*L–DOPA*_ = 28.9 ± 8.3 μV; W = 95, p = 0.002, p_*corrected*_ = 0.008, n_*dependent tests*_ = 4, BF_10_ = 3.6; rank biserial correlation = 0.62), it was evident in 25 out of 31 participants with spindle data available (Figure 5A and Supplementary material 14). This change was not correlated with the weight-adjusted L-DOPA dose (Spearman’s ρ = 0.127, *p* = 0.493, *n* = 31).

### L-DOPA’s effect on spindles is most pronounced at slow oscillation peaks

L-DOPA had a SO phase dependent effect on spindle amplitude, with a larger increase around the zero phase, at SO peak [t(30) = 2.12, *p* = 0.043, BF_10_ = 1.3]. The highest amplitude change occurred in the -π/4 to + π/4 bin, which was also the bin with the highest mean spindle amplitude for both conditions (Figures 5B, C).

To see whether this effect was specific to this spindle phase window we also performed spectral composition analyses of each SO-spindle co-occurrence. Cluster analysis of the *a priori* defined spindle phase window of 11–16 Hz, −0.5 to 0.5 s (Helfrich et al., 2018) revealed an increase in power on L-DOPA compared to placebo (*p* = 0.002; Figure 5D and Supplementary material 14). Further analyses expanding this phase window to the surrounding time-frequency space suggests that power also increases in theta (4–8 Hz) and alpha (8–12 Hz) bands following the SO peak (threshold α = 0.01, *p* = 0.002).

We found no other associations between L-DOPA and other SO characteristics (all ps > 0.09, Table 3).

**TABLE 3:** L-DOPA increases spindle amplitude.

Measure	Mean (SD)		Credible interval δ	*t*	*p*	*p* (corrected)	BF_01_
	**L-DOPA**	**Placebo**		**df = 31**			
Spindle density (# per min)	5.7 (2.2)	5.8 (4.5)	[−0.351 to 0.313]	249.0*	0.992	0.992	5.2
Spindle amplitude (μV)	28.9 (8.3)	28.3 (8.5)	[0.086 to 0.804]	95.0*	0.002	0.008	0.3**
Spindle frequency (Hz)	13.6 (0.3)	13.6 (0.3)	[−0.423 to 0.247]	197.0*	0.327	0.436	4.6
Spindle duration (sec)	0.93 (0.05)	0.94 (0.06)	[−0.636 to 0.060]	1.762	0.088	0.176	1.3
**Measure**	**Mean (SD)**		**Credible** **interval** **δ**	**t**	***p* (uncorrected)**		**BF_01_**
	**L-DOPA**	**Placebo**		**df = 30**			
**Slow oscillations**							
Slow oscillation mean amplitude (μV)	71.8 (4.9)	72.3 (4.8)	[−0.560 to 0.130]	1.280	0.210		2.5
Duration (min)	27.2 (12)	29.7 (14)	[−0.162 to 0.517]	1.083	0.287		3.1

*Wilcoxon’s test used. **Corresponding BF_10_ = 3.6. All errors < 0.055%.

### L-DOPA induced sleep changes predict memory changes

**FIGURE 6:**
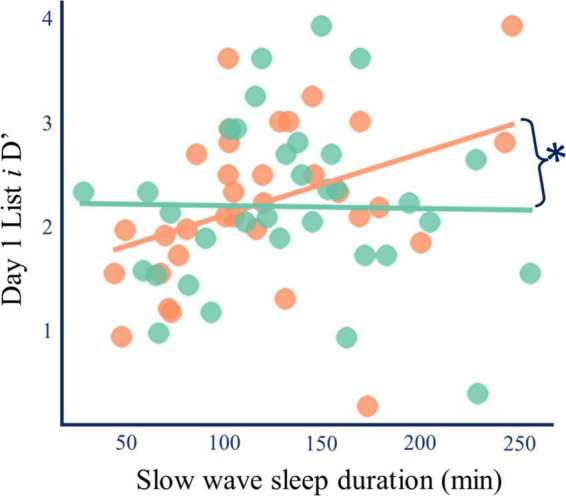
L-DOPA and slow-wave sleep duration. Longer slow-wave sleep duration was correlated with better memory for strongly encoded information on placebo (Spearman’s ρ = 0.447, *p* = 0.009, *p*_corrected_ = 0.012, orange), but after L-DOPA this relationship disappeared (ρ = 0.043, *p* = 0.810, green). The two correlations were different (r-to-z = 2.221, *p* = 0.026) (Lee and Preacher, 2013). A robustness check analysis where outlier data was excluded yielded similar results. Therefore, L-DOPA does not increase the relative effect of re-exposure by merely increasing slow-wave sleep. Lines of best fit are for illustration.

Longer slow-wave sleep duration was correlated with enhanced accuracy for the re-exposed items (List *i*) on placebo (Spearman’s ρ = 0.447, *p* = 0.009, p_*corrected*_ = 0.027, n_*dependent tests*_ = 3, *n* = 33). This effect did not occur for the single-exposure items (List *ii*), and it disappeared after L-DOPA (List *i* Spearman’s ρ = −0.043, *p* = 0.810, *n* = 33; Figure 6 and Supplementary material 15). Although L-DOPA increased both slow wave sleep duration (Figure 3) and the relative benefit of re-exposure on **d’** (Figure 2C), the paired differences (L-DOPA minus placebo) in SWS duration and re-exposure induced change in **d’** were not correlated with one another (Spearman’s ρ = −0.061, *p* = 0.744, *n* = 31).

These findings suggest that longer slow-wave sleep duration is associated with enhanced consolidation of strong but not weak memory traces, and that L-DOPA can wipe out the beneficial effect. This finding does not suggest that L-DOPA’s effect on slow-wave sleep duration explains our finding that L-DOPA reduced memory for weaker memory traces. Instead, it suggests that nocturnal L-DOPA modulates the relationship between slow-wave sleep duration and memory. Further analyses of sleep microarchitecture were performed to explore the relationship between L-DOPA, sleep, and memory.

**FIGURE 7:**
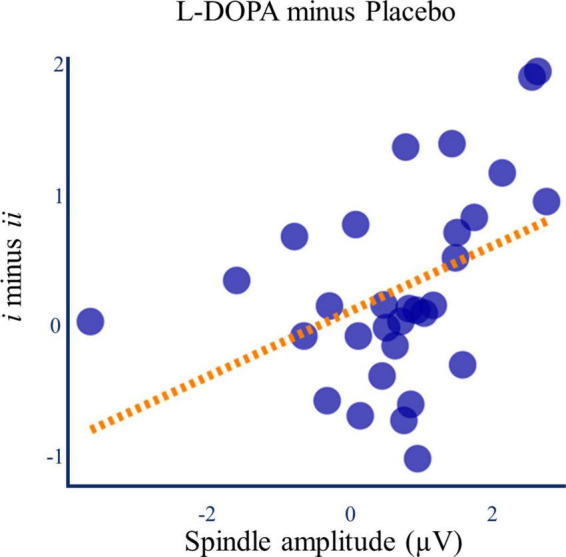
L-DOPA, memory, and spindle amplitude. The L-DOPA mediated increase in spindle amplitude was associated with the L-DOPA mediated increase in the relative benefit of re-exposure on d’ (Figure 4C) (Spearman’s ρ = 0.438, *p* = 0.015). Line of best fit is for illustration.

When looking across all spindle events, there were no correlations between spindle amplitude and the **d’** difference between Lists *i* and *ii* (L-DOPA: Spearman’s ρ = 0.047, *p* = 0.801, Placebo: Spearman’s ρ = −0.040, *p* = 0.833, *n* = 30). However, the paired change in spindle amplitude and **d’** difference for strong and weak memories between the L-DOPA and placebo nights was positively correlated (Spearman’s ρ = 0.438, *p* = 0.015, p_*corrected*_ = 0.045, n_*dependent tests*_ = 3, *n* = 30, Figure 7). This effect was specific to the L-DOPA-mediated *difference* in memory and spindle amplitude between strong and weak memory traces, and it was not present for List *i* or *ii* alone (Supplementary material 15).

The L-DOPA associated change in the ratio of non-REM 0.6–1 Hz power to 0.6–4 Hz power was not associated with L-DOPA associated changes in memory performance neither in the **d’** difference between Lists *i* and *ii* Lists (Spearman’s ρ = 0.007, *p* = 0.967) nor in the change in List *ii* performance (Spearman’s ρ = 0.150, *p* = 0.421). On L-DOPA, 1–4 Hz Slow-wave activity was correlated with worse performance on List *i* (Spearman’s ϱ = −0.460, *p* = 0.009, BF_10_ = 4.8, *n* = 31). However, this finding did not survive multiple comparison correction (p_*corrected*_ = 0.093, n_*dependent tests*_ = 10). It is possible that this analysis did not survive the correction as our study is not sufficiently powered for the relatively conservative correction approach we have adopted in this paper.

### L-DOPA does not modulate memory at encoding or retrieval—Study 2

Study 2 explored the specificity of this effect to consolidation as opposed to retrieval. In this experiment, we administered short-acting L-DOPA before encoding (encoding condition) or retrieval (retrieval condition) to specifically target dopamine’s action to those processes. L-DOPA had no effect on encoding [paired t(28) = −0.352, *p* = 0.728, BF_01_ = 4.6] or retrieval [t(27) = −0.393, *p* = 0.698, BF_01_ = 4.6, for full results, see Supplementary material 16]. Therefore, we found no effect of L-DOPA on encoding or retrieval in an experimental setting similar to that of the main study, suggesting the results reported above are unlikely to be due to L-DOPA’s action during encoding of List *ii* during repeat exposure or during retrieval due to the possibility that residual amounts of L-DOPA were present in the system during the recognition memory tests for Lists *i* and *ii.* Whilst the results from this control study support our initial interpretation that L-DOPA affects memory *after* initial learning but *before* retrieval, it is important to note that, due to the different doses and timings used here, direct statistical comparisons between the two studies cannot be made.

## Discussion

We report findings from two clinical trials of L-DOPA in older people, designed to tease apart the temporal relationships between L-DOPA administration, sleep and memory. L-DOPA accelerated forgetting/memory loss for weakly encoded information during sleep—while more strongly encoded information was preserved. Physiologically, L-DOPA increased slow-wave sleep duration by 10.6%, increased spindle amplitude around SO peaks and increased 1–4 Hz slow-wave power. Increased spindle amplitude on L-DOPA correlated with the difference between the effect of L-DOPA on strong compared to weak memory traces. About 1–4 Hz slow-wave power correlated with loss of memory (or forgetting) for words overnight. Overall, our data point toward a role for dopamine in memory selection during sleep.

Traditionally, forgetting is considered a passive process where information is “lost.” However, newer animal models support an active, more strategic, forgetting process mediated by dopamine (Davis and Zhong, 2017). In drosophila, several studies have shown that dopamine is involved in strategic forgetting (Berry et al., 2012, 2018; Sabandal et al., 2021), and that sleep facilitates this effect (Berry et al., 2015). Supporting evidence from rats has shown that dopamine is necessary for memory destabilisation after re-exposure (Rossato et al., 2015; Gonzalez et al., 2021). This de-stabilisation can help shape existing memories by integrating them with novel information, but it also makes memories more vulnerable to forgetting. Here, the L-DOPA-driven bias away from retaining weaker memory traces was associated with *increased* spindle amplitude, supporting an *active* dopamine-dependent forgetting mechanism in humans.

While changes in spindle characteristics are well known to be associated with memory and neurodegeneration (Latreille et al., 2015), this study directly links the dopamine precursor L-DOPA with the behavioural relevance of spindles in elderly individuals. Spindle amplitude is shaped by the interplay between the thalamus and the cortex (Contreras et al., 1997), and increased amplitude reflects better coordination of spindle-related corticothalamic networks (Nir et al., 2011; Cox et al., 2017). Behaviourally, increased spindle amplitude has been associated with enhanced memory retention (Heib et al., 2015; Blaskovich et al., 2017). This coordinated activity between the thalamus and cortex during sleep may thus be associated with selecting memories for later retention or forgetting, and this may be augmented by dopamine before or during sleep.

L-DOPA mainly increased spindle amplitude around the peak of SOs, which occurred despite no change in SO amplitude. Spindles, particularly when nested in SO peaks, are considered hallmarks of sleep-dependent memory consolidation (Staresina et al., 2015). Uncoupling of spindles and SO peaks is associated with increased overnight forgetting in older people (Helfrich et al., 2018). Considering that L-DOPA increased forgetting only for weak engrams, we interpret our findings as revealing L-DOPA-induced enhancement of physiological spindle-slow-oscillation coupling to bias memory selection.

L-DOPA also increased power in the 1–4 Hz band during non-REM sleep. The balance between 1 and 4 Hz activity and <1 Hz SOs has itself been proposed as a key determinant in the fate of information, with SO/spindle concordance-driven long-term memory persistence countered by 1–4 Hz activity attenuating reactivation strength and biasing toward forgetting (Mander et al., 2015; Kim et al., 2019; Klinzing et al., 2019; Ngo and Born, 2019).

The effect of dopamine on memory could take place either during sleep to increase spindle amplitude which in turn biases memory based on encoding strength, or it could act before sleep (during memory re-exposure and/or shortly afterward) with a secondary effect on sleep. These scenarios are not mutually exclusive, and indeed could be interacting. Furthermore, as we did not have a wake comparison condition, we cannot ascertain that the effects on memory are truly sleep-specific.

It is possible that our re-exposure condition acted as retrieval-mediated learning due to the recognition memory component of the evening re-exposure of List *i*. Memories that have been practiced by retrieval are reinforced and it has previously been suggested that retrieval practice may act as a fast lane to memory consolidation by activating electrophysiological events that parallel those seen during sleep (Antony et al., 2017). Others have shown that there is no further added benefit of sleep on the persistence of memories that have been practiced by retrieval (Bauml et al., 2014; Antony and Paller, 2018). Therefore, although we report disparate sleep microarchitectural effects associated with strong and weak memories, we cannot exclude that the disparate effects in the current study are explained by fast-paced consolidation during wakeful retrieval-practice rather than memory strength.

Note that there is one further caveat to interpreting our re-exposure data which is that re-exposure occurred on L-DOPA (as our original intention was to isolate learning from the effects of L-DOPA—to show that the L-DOPA has an effect after initial learning). However, this means we do not know whether the effect of L-DOPA during re-exposure or later, during consolidation and/or sleep. The sleep EEG changes on L-DOPA suggest that L-DOPA has an impact beyond the moment of re-exposure, but this will require further testing to be definitive. Future experiments are necessary to disentangle these options.

We did not show any direct benefit of post-encoding L-DOPA on remembering of re-exposure information. At face value, this is at odds with the Synaptic Tagging and Capture theory which predicts that within a few hours of encoding, dopamine should stimulate the synthesis of Plasticity-Related Proteins that in turn consolidate the Synaptic Tags created by prior memory encoding, leading to enhancement of memory persistence (Takeuchi et al., 2016). Whilst we did not find a benefit of L-DOPA on retaining information, we did find that it only increased forgetting of *weak* engrams.

Therefore, two simultaneous processes may be at play. First, while we did not find evidence to support this, previous studies suggest that during learning a portion of information is “tagged” as important (Frey and Morris, 1998), and dopamine enhances this process by creating a stronger tag (Sajikumar and Frey, 2004; Clopath, 2012). Second, during sleep, dopamine may accelerate forgetting for the less important, or non-tagged, items while the tag shields important information from forgetting (Rauchs et al., 2011; O’Donnell and Sejnowski, 2014). This increased forgetting might be driven either by dopamine-mediated crosstalk between the thalamus and the cortex during spindles, or by dopamine-mediated increase in non-REM 1–4 Hz spectral power. In support of this interpretation, stimulation paradigms mimicking slow-wave activity induce long-term synaptic depression in somatosensory pathways (Pigeat et al., 2015). Such synaptic downscaling in restoring homeostasis for new memory acquisition offers a plausible explanation for weaker engram loss (Ribeiro, 2012).

**FIGURE 8:**
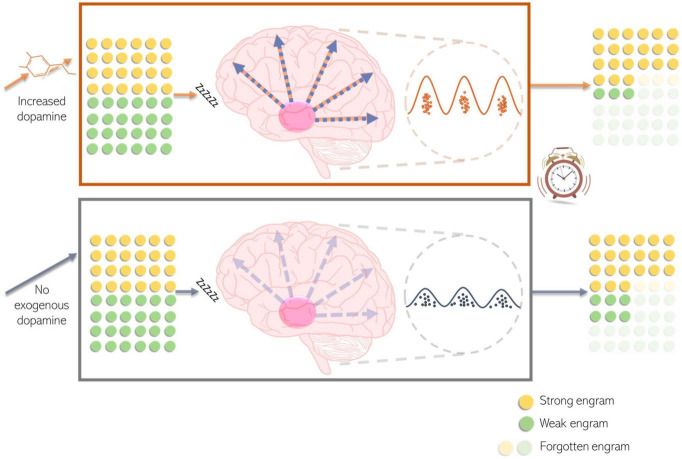
Model of dopamine modulation of memory. Some routine information forms a stronger (yellow) memory engram than other routine information (green), for example due to re-exposure or salience. While capacity for overall consolidation remains the same, an increase in dopamine availability causes destabilisation and preferential forgetting of weak engrams. Dopamine modulates this memory selection by enhancing synchronisation in cortical firing patterns during spindles, at the peak of slow-waves. Together these two processes (enhanced forgetting and cortical synchronisation) bias subsequent memory.

Therefore, we propose a model (Figure 8) that, rather than being at odds with previous literature, emphasises the simultaneous role of post-encoding dopamine in memory selection by enhancing forgetting for weak engrams, possibly via its effects on slow wave sleep.

We showed that higher L-DOPA dose was associated with increased forgetting for weak engrams. However, we found no effect of dose on L-DOPA’s effects on slow wave sleep duration. L-DOPA dose effects are particularly important to explore given that previous studies (Morcom et al., 2010; Chowdhury et al., 2012) have shown disparate effects of exogenous dopamine on memory depending on dose. We did not replicate this finding here, possibly due to our weight-adjusted dose window being too narrow, as all participants were of similar body weight. This might also explain why the magnitude of increase in slow wave sleep duration was not associated with dose, although L-DOPA compared to placebo increased duration of this sleep stage.

Dopamine’s effects on cognitive function have been stipulated to follow an inverse U-shaped curve (Cools and D’Esposito, 2011), where both too much and too little dopamine activity can impair cognition. Indeed, in similar paradigms to ours dopamine-like medication has been associated with changes in enhanced memory persistence in healthy elderly (Grogan et al., 2015) in a dose-dependent fashion (Chowdhury et al., 2012), supporting the inverted U-shape hypothesis. Yet, others have found no impact of a dopamine agonist on verbal memory persistence (Feld et al., 2014), or a dopamine d2-like receptor blocker (Asfestani et al., 2020) on overnight reward consolidation in young adults. These differences in findings may be due to different medications or due to age-related reductions in dopamine neurons (Carlsson et al., 1980; Kish et al., 1992; MacDonald et al., 2012) and/or slow-wave sleep duration, spindle counts and amplitudes (Nicolas et al., 2001).

In line with our results, previous studies have shown that selective neurotoxicity to dopamine neurons causes a dose-dependent decrease in non-REM sleep in mice (Gerashchenko et al., 2006; Revishchin et al., 2016). However, in humans dopamine antagonism has been shown to increase non-REM duration (Eder et al., 2003). Whilst at face value this appears to be at odds with our finding that L-DOPA increases slow wave sleep duration in elderly, others have shown that dopamine may have a biphasic, dose-dependent effect on sleep. Specifically, lower doses may increase non-REM sleep and low frequency band power density, with most pronounced increase in SO activity, while higher doses are associated with the opposite effect (Monti et al., 1988; Kropf and Kuschinsky, 1991; Sebban et al., 1999). Therefore, our finding that L-DOPA increases slow wave sleep duration should be carefully interpreted to only apply to our current dosing regimen.

Overall, we show that L-DOPA plays an important role in memory selection, mediated by increased slow-wave sleep duration and spindle amplitude. We propose that this L-DOPA-induced increase in spindle amplitude reflects more synchronous cortical activity during spindles increasing forgetting of weakly encoded items and with a net effect of augmenting the difference between strongly and weakly encoded engrams. These findings have potential clinical impact for development of strategies for modulating sleep to prevent neurodegeneration and enhance cognition in later life.

## Data availability statement

The original contributions presented in this study are included in the article/Supplementary material, further inquiries can be directed to the corresponding authors.

## Ethics statement

The studies involving human participants were reviewed and approved by South-West 3 Research Ethics Committee. The patients/participants provided their written informed consent to participate in this study.

## Author contributions

HI and EC designed the Study 1. MJ, CD, CO’D, and LM contributed significantly to designing Study 1. LM performed randomisation and blinding for Study 1. HI, JG, and EC designed the Study 2 and oversaw all data collection. HI and JG developed the verbal memory tasks. HI developed the visual/reward memory task. HI, GA, JB, JG, WC, and UB wrote all analysis scripts. WC and OR performed the sleep scoring which was overseen by HI. HI, WC, JB, and UB carried out the spindle and SO analyses. CO’D and UB gave further statistical guidance. HI, WC, GA, OR, JS, RW, EF, JM, CD, AW, CM-N, and JG collected the data. EC, JM, and JS provided further clinical cover. HI, EC, WC, JG, CO’D, MJ, and UB interpreted the data. HI, EC, and JB wrote the manuscript. All authors contributed to the editing of the manuscript and approved of the final version.
