# Corrigendum: L-DOPA increases slow-wave sleep duration and selectively modulates memory persistence in older adults

**DOI:** 10.3389/fnbeh.2024.1478382

**Published:** 2024-08-28

**Authors:** Hanna K. Isotalus, Will J. Carr, Jonathan Blackman, George G. Averill, Oliver Radtke, James Selwood, Rachel Williams, Elizabeth Ford, Liz McCullagh, James McErlane, Cian O'Donnell, Claire Durant, Ullrich Bartsch, Matt W. Jones, Carlos Muñoz-Neira, Alfie R. Wearn, John P. Grogan, Elizabeth J. Coulthard

**Affiliations:** ^1^Clinical Neurosciences, Translational Health Sciences, Bristol Medical School, University of Bristol, Bristol, United Kingdom; ^2^Digital Health, Faculty of Engineering, University of Bristol, Bristol, United Kingdom; ^3^Southmead Hospital, North Bristol NHS Trust, Bristol, United Kingdom; ^4^Department of Neurosurgery, Heinrich-Heine-University Clinic, Düsseldorf, Germany; ^5^Production Pharmacy, Bristol Royal Infirmary, University Hospitals Bristol and Weston NHS Trust, Bristol, United Kingdom; ^6^School of Computer Science, Electrical and Electronic Engineering, and Engineering Mathematics, University of Bristol, Bristol, United Kingdom; ^7^Experimental Psychology, University of Bristol, Bristol, United Kingdom; ^8^School of Physiology, Pharmacology and Neuroscience, University of Bristol, Bristol, United Kingdom; ^9^Nuffield Department of Clinical Neurosciences, University of Oxford, Oxford, United Kingdom; ^10^School of Psychology, Trinity College Dublin, Dublin, Ireland

**Keywords:** sleep, memory, dopamine, ageing, slow wave sleep, NREM, levodopa, learning

In the published article, there was an error in the **Funding** statement. The Doctoral Training Grant number was erroneously omitted. The correct **Funding** statement appears below.

