# Supplementary material for: L-DOPA increases slow-wave sleep duration and selectively modulates memory persistence in older adults
Source: Front Behav Neurosci. 2023 Apr 5;17:1096720. doi: 10.3389/fnbeh.2023.1096720 (PMC10113484; doi:10.3389/fnbeh.2023.1096720)
Supplement: Supplementary file 1 [file Data_Sheet_1.PDF]

Supplementary Material

1 SM1 : Full method

All aspects of this research adhered with the Declaration of Helsinki and we had relevant ethical and regulatory (UK) approvals in place (Study 1 ISRCTN: 90897064).

Design

Study 1: In the main placebo-controlled double-blind randomised study, volunteers were initially screened over the phone for common exclusions, and then invited for three in-house visits. On the first visit they were fully screened for eligibility, and they practiced the memory task. They were asked about their usual sleeping pattern so that the second and third visits could be designed to follow each participants' usual sleep routines as much as possible. On the second visit, volunteers arrived on site in the evening where they were re-consented and screened for continued eligibility. For an outline of the evening see **Fig. 1a**.

First, volunteers learnt a verbal memory task (**Fig. 1b.**). Thirty minutes after learning, they were given 200mg L-DOPA or placebo. After a 75 min interval following dosing, a quarter of the items (List *i*) were re-exposed by a recognition test where no feedback was given. The purpose of this test was to create a stronger memory trace. After a 45 min following the re-exposure the volunteers went to bed. Each evening was designed based on each participants' usual sleeping pattern (L-DOPA administered 2h prior to switching the lights off for the night at their usual bedtime).

Volunteers slept on-site for a full night, and they were woken up at their usual wake-up time. Around 1.5h after waking up, approximately 12h after dosing, volunteers' verbal memory was tested again (Lists *i* and *ii)* before they left the study site. 2 and 4 days later (3 and 5 days after learning) they were contacted over the phone for follow-up recognition memory tests (for Lists *iii* and *iv*, respectively).

The second and third visits were identical except for treatment (L-DOPA / placebo) allocation. This study obtained ethical approval from the South West Central Bristol NHS Research Ethics Committee (REF: 16/SW0028) and clinical trial authorisation from the Medicines and Healthcare products Regulatory Agency (IRAS ID:178711) .

Study 2: In the secondary placebo-controlled double-blind crossover experiment, volunteers were first screened over the phone before inviting them on site for the test sets. Each test set carried over for three days: On the Day -1 (relative to dosing) participants learnt word list on site, on Day 0 they were dosed with 150mg L-DOPA (or placebo) and then tested on the previously learnt words (retrieval test). Immediately following this, they learnt another word

list (encoding condition) for which their memory was tested the following day (Day 1) over the phone. Therefore, participants learnt two word lists on each test These tests were timed so that there was approximately 24h in between learning and test.

This study obtained ethical approval from the University of Bristol Faculty of Medicine and Dentistry Ethics Committee (REF: 12161).

Treatment

Study 1: In the main placebo-controlled randomised double-blind study, each participant was dosed with co-beneldopa controlled release (containing 200mg L-DOPA) given in capsule form and placebo (encapsulated inert powder, matched for appearance). Blinding and randomisation were performed in blocks of 6 by author LM, Production Pharmacy, Bristol Royal Infirmary, University Hospitals Bristol and Weston NHS Trust. On the study nights, dose was given by an on-site medic who was blind to treatment condition and played no role in collecting data. The treatments were given at different visits. Both treatments were preceded by Domperidone 10mg (tablet) to alleviate possible nausea caused by L-DOPA.

Study 2: In the L-DOPA condition of the secondary study volunteers received 10mg of Domperidone (anti-emetic) 30 minutes before co-beneldopa (containing 150mg L-DOPA). Both medications were dispersible, and they mixed into cordial to hide taste and residue. In the placebo condition, volunteers received plain cordial in place of Domperidone and vitamin C mixed into cordial in place of L-DOPA. Blinding and randomisation were performed by members of the research group who had no other involvement in this study.

Verbal memory test

Study 1: Volunteers learnt four lists (*i, ii, iii,* and *iv*) of 20 target words (total 80 targets) presented on a computer screen one at a time, in a random, interleaved order (**Fig. 1b.,** *SM 12*). Each word was presented once for 3.6s during which the volunteers were asked to determine if the items were alive or not to assist learning. They were instructed to remember as many of the words as they could.

During test phases, volunteers were presented with a list of 40 (days 0, 3, and 5) or 80 words (day 1), half of which were targets (present at learning) and half of which were distractors (not presented previously). They were asked to judge whether words were targets or not. On days 0, 1, 3, and 5 memory was tested for Lists *i*, *i* and *ii*, *iii*, and *iv* respectively. Therefore, List *i* was tested twice: First in the evening while L-DOPA (/ placebo) was active in the system and then again in the morning together with List *ii*. The re-exposed and novel (List *i* and List *ii*, respectively) targets tested on day 1 were assessed to study L-DOPA's effect on behavioural tagging of 'important' information. The rationale was that when a word is presented a second

time (during re-exposure), it will be deemed more important and will be preferentially remembered. The distractors were unique at each test.

Study 2: The purpose of this study was to test L-DOPA's effects on retrieval and encoding. Two separate memory tests were conducted (*SM 13).*

Retrieval: During learning on D-1 (day before dosing) volunteers were presented with 48 complete nouns on a computer screen. They were instructed to read the words aloud and try to memorise them for later. Each word was shown once for 5 seconds separated by a fixation cross in the middle of the screen for 2 seconds and no responses to the words were made during learning. There were no breaks in the learning block (total duration = 5mins 36secs). Memory was tested using unique words 30 minutes (D-1, baseline) and 24 hours (D0) after learning. The D0 test was given when L-DOPA was at its peak concentration (~ 1h following dosing). This test took approximately 5 minutes to administer.

Encoding: D0 around 1.5 hours after dosing, after the test for the previous task had finished, volunteers saw a list of 96 complete nouns presented on the computer screen. Each word was displayed for 5 seconds, followed by a fixation cross for 2 seconds. The words were first presented in a random order over two blocks, and then again in a different random order over two more blocks, each word was presented twice (n blocks = 4, n words per block = 48, n breaks = 3, block duration = 5 min 36 s). Memory was prompted immediately after learning (D0), and 1, 3, and 5 days later. Each target was tested once with unique distractors (*SM 14*).

Across experiments, learning and tests were completed on a laptop on-site, or over the phone. The experiments were programmed in the MATLAB environment (2015b or 2017a) using the Psychophysics Toolbox V3 [1]. The scripts and data are available from corresponding authors upon request.

Polysomnography

In the main experiment, standard in-laboratory polysomnography, including video, was recorded during both study nights using the Embla N9000 amplifier and Embla RemLogic software (Natus Medical Inc., California) at CRIC Bristol, University of Bristol, Bristol, UK. We recorded 12 scalp EEG channels (F3, Fz, F4, C3, Cz, C4, M1, Pz, M2, O1, O2, and a ground electrode approximately between Cz/P3 and C3/Pz) placed according to the 10-20-20 system. Eye movements were detected by electro-oculogram recorded from E1 and E2 sites, and muscle tone from electromyogram recorded below the chin. A 2-lead ECG was also recorded. All signals were sampled at 500Hz. The recordings started 2.5h after dosing when lights were switched off for the night and continued until the volunteer woke up.

Analyses - EEG

Event scoring: Sleep stages in 30s epochs were identified manually in accordance with standard criteria [2] . Scoring was split between two expert scorers, with a third scorer visually assessing a random 10% of ratings for quality assurance. Durations of N1, N2, N3 (i.e. slow wave sleep), REM, awake, asleep and total time in bed were extracted in minutes. First and second halves of the nights were defined by the middle time-point between switching lights ON and OFF. When there was an odd number of epochs, they were rounded so that the first half of the night had the extra epoch.

Spindle detection: Spindle events were then isolated with in-house written MATLAB scripts using the EEGlab toolbox [3] using similar criteria to previous studies [4,5]. Electrodes were re-referenced to contralateral mastoid and empty and high variance epochs were removed. Thereafter, only data from the Cz electrode was used. First, the channel was visually inspected and epochs with high noise or clear artefacts were removed manually. Data was then filtered (high pass 11Hz, low pass 17Hz) and rectified. Next data was smoothed using a moving average window of 200ms before down-sampling to 100Hz (from 500Hz) for computational efficiency. An event was marked as a spindle if the threshold exceeded the 90th percentile for that data set (i.e. sorting data into an ascending order and including top 10%) for .5 – 3 seconds, with a minimum of 0.5s between spindle events. The threshold was chosen based on visual inspection of detected events and order of magnitude checks of quantities of spindles detected.

Slow oscillations: The slow oscillation detection process followed the same re-referencing and noise removal methods used for spindle detection, without smoothing, again in accordance with previous studies [4,5]. Data from the CZ electrode was filtered between 0.16Hz and 1.25Hz and then z-scored. We applied a threshold of 75%; if the slow oscillation amplitude surpassed this threshold for 0.5 - 5 seconds (including multiple events if separated by <0.25s), it was marked as a slow oscillation. The duration of the event was determined by the closest oscillation maxima following the amplitude dropping below a 60% threshold on each side. Thresholds were chosen in a similar fashion to spindle events.

Spindles and slow oscillations: We identified spindle-slow oscillation co-occurrences as cases where the maximum amplitude of a spindle event coincided with a slow oscillation event, again using the CZ electrode. Using the time stamp of the spindle max amplitude as the centre point, we calculated how spindle amplitude varied with slow oscillation phase over one cycle. First, we divided the oscillation events into 16 bins, equally distributed in phase space around zero, to calculate how the spindle amplitude varied with slow oscillation phase for each coinciding case (for statistical analysis we grouped together 4 adjacent frequency data points [bins] to generate 4 bins as shown in **Fig. 4c**).

The spectral composition of each was done using a Morlet wavelet time-frequency method over a 4s window centred on the slow oscillation peak. Morlet waves at 20 frequencies were used, with 3 cycles for the lowest frequency (1Hz) and 6 cycles otherwise (2-20Hz). A spectral mean

was next calculated for each participant for L-DOPA and placebo conditions (**Fig. 4e.**). A cluster-based permutation method (permutation n = 500, cluster threshold of α = 0.01), implemented with the Fieldtrip toolbox [6], was used to identify power differences. Based on the finding that max spindle amplitude occurring near zero slow oscillation phase predicts to memory performance in ageing [7], an a-priori spindle region of interest of 11 - 16Hz, -0.5 - 0.5s was chosen for initial analysis. This same cluster method was then carried out on the wider time-frequency space, 1 – 20Hz, -1 – 1s, the primary cluster (p=0.002) of which is shown in **Fig. 4e.**

Analyses - Behaviour

Pairwise comparisons (placebo versus L-DOPA) were calculated using either t-tests or Wilcoxon's rank tests in R 3.5.3 using RStudio. We also employed a Bayesian paired t-tests in JASP 0.9.2.0 [8] to obtain Bayes Factors (BF) – this allows more meaningful estimates of confidence in both significantly different and null results than standard t-tests. BF gives the probability of the data under either hypothesis. E.g. a BF_{10} of 5 would denote that the data is 5 times more likely to have been sampled from the alternative compared to the null distribution, while a BF_{01} of 5 would denote that the data is 5 times less likely to have been sampled from the alternative compared to the null distribution (i.e. 01 versus 10). We defined the prior (expected) distribution as a Cauchy distribution with a mean of 0 and an interquartile range of .5 [$\delta \sim$ Cauchy (0, .5)]. In other words, we predicted that the δ lies between -.5 and .5 with a 50% confidence. We selected this one as the δs in cognitive neurosciences typically are within those bounds, and as we did not have an informed prediction for the effect sizes.

All mixed modelling was performed on R 3.5.3 using Rstudio, lme4 [9] and lmerTest [10]. We included the participants as random effects and the dose (mg/kg) and the memory test delay (Day 1, Day 3 and Day 5), or memory strength (re-enforced versus not), depending on the analysis, as fixed effects. All fixed effects were mean-centred but not scaled. We selected the model using the maximum feasible fit as this has previously been shown to be the best approach for confirmatory hypothesis testing [11].

2 SM2 : Inclusion and exclusion criteria

Inclusion The inclusion criteria for Studies 1 and 2 were the same; volunteers were over 65 years, native or fluent English speakers, and had normal or corrected-to-normal vision allowing them to read text on a computer screen.

Exclusion

Participants did not have:

- clinically significant neurological or psychiatric diagnoses as assessed by self-report and questionnaires during the screening visit.
- a diagnosis of mild cognitive impairment or dementia.
- undiagnosed skin lesions.
- sensitivity to levodopa, benserazide, or domperidone.
- lactose intolerance, galactosemia or glucose/galactose malabsorption.
- galactose intolerance.
- Lapp lactase deficiency.
- diagnosis of Huntington's Chorea.
- clinically significant intention tremor.
- known prolactin-releasing pituitary tumour (prolactinoma).
- diagnosis of glaucoma.
- a history of, or current, malignant melanoma.
- current cancer treatment.
- diagnosed unstable diabetes (people with stable type 2 diabetes diet-controlled diabetes were included)
- severe endocrine, hepatic, renal, pulmonary, or cardiac disorder.
- diagnosed electrolyte disturbances.
- known peptic ulcers.
- history of a heart-attack or prolongation of cardiac conduction intervals, or any other cardiac problems as taking domperidone increases risk of said problems.
- childbearing potential or pregnancy.

Participants were also excluded if they were taking any of the following:

- dopaminergic medications.
- noradrenergic, serotonergic, or anticholinergic medications started or changed within the past 3 months.
- monoamine oxidase inhibitors (MAO-I), except if selective MAO-A or MAO-B inhibitors are given alone. MAO-A and MAO-B inhibitors given together are equivalent to non-selective MAO-inhibition and therefore volunteers taking both MAO-A and MAO-B were not included.
- cholinesterase inhibitors, except if the participant was on stable treatment (at least 3 months).
- antihypertensives containing reserpine.
- ferrous sulphate on the day of dosing.

- opioids or sympathomimetics unless if the participant was able to abstain on the day of dosing.
- diazepam or other benzodiasepines, unless none taken for prior 3 days or stable dose was maintained for more than 3 months.
- ketoconazole, erythromycin or CYP3A4 inhibitors
- antibiotics, if taken to treat an active infection.
- hormone replacement therapy.
- anti-fungal agents (pentamidine).
- anti-malarial agents.
- antihistaminics unless stable dose for 3 months or none for 3 days prior to testing sessions.
- AIDS/HIV medications.
- Any QTc prolonging medicinal products.
- if a participant took antacids or antisecretory agents they were required not to be taken at the same time as domperidone

Additional exclusion criteria for study 1

For Experiment 1, we also excluded volunteers with clinically significant sleep problems in the past year. We consider a sleep disorder to be clinically significant when it results in fewer than 6 hours' sleep per night regularly, in the estimation of the volunteer, and they perceived to have impaired sleep. We also excluded sleep disorders that required intervention (including equipment or medication) likely to interfere with our protocol or people with diagnosed sleep disorders who require intervention but who are unable to or have decided not to have the intervention (e.g. people with sleep apnoea for which a mask was recommended but who could not tolerate the mask). We also excluded one volunteer for being a wheelchair user, as we could not accommodate for a carer to stay with the volunteer.

frontiers

3 SM3 : Demographic information

	Encoding (Study 2) *n= 32*		Consolidation (Main study / Study 1) *n = 35*			Retrieving (Study 2) *n = 28*	
	**Mean** (SD)	**Range**	**Mean** (SD)	**Range**		**Mean** (SD)	**Range**
**Age**	**71.1** (7.1)	65 – 92	**68.9** (3.5)	65 – 79		**70.9** (6.9)	65 – 92
**Years of education**	**14.7** (3.5)	10 – 24	–	–		**14.5** (3.5)	10 – 24
**Montreal Cognitive Assessment**	**26.1** (3.3)	18 – 30	**27.5** (2.5)	21 – 30		**26.1** (3.2)	18 – 30
**Height (cm)**	**170.0** (10.3)	152 – 186	**166.1** (7.4)	152 – 181		**170.0** (10.6)	152 – 186
**Weight (kg)**	**75.2** (15.4)	51 – 105	**70.28** (13.0)	48 – 94		**75.0** (15.6)	51 – 105
**Body mass index (kg/m^2)**	**25.8** (3.7)	18.1 – 35.1	**25.2** (3.2)	18.5 – 32.7		**25.8** (3.7)	18.1 – 35.1
**L-DOPA concentration (mg/kg)**	**2.08** (0.44)	1.43 – 2.95	**2.94** (0.54)	2.13 – 4.17		**2.09** (0.45)	1.43 – 2.95
**Gender (f/m)**		**16** / **16**		**22** / **13**			**14** / **14**
**Treatment order (L-DOPA/ Placebo first)**		**17** / **15**		**18** / **17**			**13** / **15**
**Blinding (accurate / inaccurate** / missing)		**17** / **12** / 3		**21** / **13** / 1			**16** / **10** / 2
**Rationality Experientiality index (REI) scores**							
Overall rationality	**3.6** (0.8)	1.5 – 5.0	**3.5** (0.7)	2.3 – 4.7		**3.7** (0.9)	1.5 – 5.0
Rational Engagement	**3.8** (0.9)	1.0 – 5.0	**3.4** (0.7)	2.1 – 4.6		**3.8** (0.9)	1.0 – 5.0
Rational Ability	**3.5** (1.0)	1.0 – 5.0	**3.5** (0.7)	2.1 – 4.7		**3.5** (1.0)	1.0 – 5.0
Overall Experientiality	**3.1** (0.6)	2.0 – 4.2	**3.4** (0.7)	2.1 – 5.0		**3.1** (0.6)	2.0 – 4.2
Experiential Engagement	**2.9** (0.7)	1.0 – 4.2	**3.3** (0.6)	2.2 – 5		**2.9** (0.8)	1.0 – 4.2
Experiential Ability	**3.3** (0.6)	2.0 – 4.5	**3.5** (0.7)	1.9 – 4.9		**3.3** (0.6)	2.0 – 4.3
**Depression, Anxiety and Stress Scale (DASS)**							
Depression	**4.2** (4.9)	0 – 18	**2.1** (3.2)	0 – 11		**4.0** (4.7)	0 – 18
Anxiety	**2.0** (2.4)	0 – 11	**1.8** (2.3)	0 – 8		**2.0** (2.6)	0 – 11
Stress	**5.4** (4.1)	0 – 14	**6.2** (5.9)	0 – 21		**5.4** (4.3)	0 – 14
**Barratt Impulsivity Scale (BIS)**							
Motor impulsiveness	**21.3** (3.7)	14 – 29	**21.1** (3.6)	12 – 27		**20.9** (3.5)	14 – 29
Non-planning	**22.1** (5.1)	11 – 30	**21.7** (5.3)	14 – 35		**21.6** (4.7)	11 – 30
Attentional	**14.6** (2.5)	10 – 19	**14.5** (3.4)	9 – 23		**14.4** (2.5)	10 – 19
**Pittsburgh sleep quality index**		–	**35** (10.4)	20 – 59		–	–
Sleep efficiency	–	–	**1.4** (1.5)	–	–	–	–
Sleep Quality	–	–	**1.7** (1.6)	–	–	–	–
Daily disturbance	–	–	**1.4** (0.7)	–	–	–	–

Demographic variables.

For the main study (middle column) BIS was missing from one volunteer. Otherwise, full datasets for participants who completed each experiment are reported. L-DOPA concentration was calculated as drug dose (mg) / body weight (kg). In Study 2, volunteers were dosed with 150mg and in Study 1 with 200mg L-DOPA CR, both in the form of co-beneldopa.

4 SM4 : Verbal memory task in Study 1

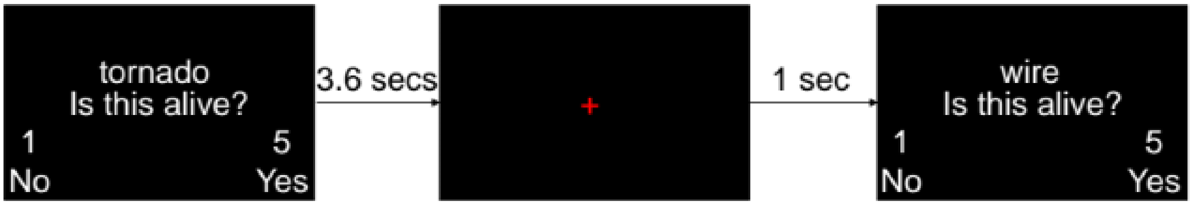

Supplementary Figure: In this task, volunteers first learnt a list of 80 words presented on a computer screen in a random order, one at a time. While each word was presented, volunteers performed an incidental task where they were asked to determine if the items were 'alive' or not by pressing one of two buttons on a custom-made response box (https://www.blackboxtoolkit.com). Each word was displayed once for 3.6s, whether a response was made or not, and separated by a 1s fixation cross. Words were separated into two 40-word blocks to allow a break.

In the test phase of the tasks, volunteers were shown a list of words presented individually, half of which were targets (present at learning) and half of which were distractors (not present at learning). They were asked to judge whether they had seen a word previously by judging the word as 'OLD' (target) or 'NEW' (distractor). On Days 0, 3, and 5, participants were shown 20 targets and 20 distractors, and on Day 1, 40 targets and 40 distractors were shown. The same targets as were present on Day 0 were re-tested on Day 1 (i.e. they were biased as behaviourally salient by re-exposure), together with targets that had only been seen during learning and not during day 0 testing (non-salient targets). Each distractor was only seen once. The exposed and re-exposed targets were compared to study L-DOPAs effect on behaviourally "tagging" important memories with the rationale that when a word is presented a second time by a recognition test, it will be deemed to be more 'worthy' of being remembered later than a word that is presented once.

All on-screen text was displayed using Helvetica font (red on white background) on a Toshiba laptop. On days 0 and 1 testing was completed on a laptop, on days 3 and 5 testing was completed over the phone.

After each 'OLD' / 'NEW' judgement volunteers completed remember-know judgements on the items [12]. The experiment was programmed in the MATLAB environment (2015b or 2017a) using the Psychophysics Toolbox V3 [1]. The full script, standard operating procedures for administering the task, and task instructions are available from the corresponding author upon request.

5 SM5 : Reward memory task

Participants completed a visual memory task with a reward element in this study. The purpose of this task was to assess dopamine's effect on reward memory. However, the reward manipulation was not successful and therefore this experiment was not further analysed. In other words, the experimental manipulation was not sufficient to observe an effect. A brief description is given below.

Learning: During learning, participants were shown two images presented together and their task was to memorise figure pairs. Before each pair was presented, participants saw a reward – either a high reward (stash of £20 notes equating to ~£1000) or a low reward (a handful of coins equating to £4) – displayed on the screen. They were instructed that their subsequent memory performance would be scored based on the value shown before the trial (some pairs gained them a higher 'memory score' than others) but that they did not need to pay it undue attention.

After the reward was shown, a pair of figures was shown on the screen; one on the top of the screen and another in the bottom of the screen shown in one of three locations in the bottom of the screen (left, middle, or right). Participants were asked to memorise which figures were shown together and in which location the bottom figure was shown in. To enhance learning, we asked participants to also imagine the two figures (e.g. a giraffe and a roller pin) interacting in some way (i.e. "For example, you could imagine the giraffe baking a cake using the roller pin"). To ensure participants were paying attention, they were also asked to indicate in which of the three locations the bottom figure was in using a custom-made response pad with three horizontally aligned round buttons. After pressing the button, a blue circle appeared on the screen around the location they had indicated.

Therefore, participants had to remember which figures were shown together and in which of the three locations the bottom figure was given in. Half of the pairs were associated with higher value than the other half.

All pairs were first presented in a random order over two learning blocks, and then again in another random order over two more learning blocks. Between each trial, participants saw a fixation cross for 2s, followed by the reward for 1s, and then immediately they would see the pairs on the screen for 7.5s (n pairs = 40, n trials = 80, n high reward pairs = 20, n low reward pairs = 20, n blocks = 4).

Memory test: Memory for the items was tested the following morning. During the test, participants saw one figure on the top of the screen with three figures in the bottom of the screen. During learning, the figure at the top had been shown in the bottom of the screen, and each of the figures in the bottom had been shown on the top of the screen but during different learning trials. During the first half of the test, participants were asked to indicate the correct pair for the top figure using a button box. During the second half of the test, the bottom figure was shown in each of the possible locations and participants were asked to indicate where the figure was shown during learning.

The following morning (L-DOPA no longer active) all pairs were tested.

Summary of results: We expected that the highly rewarded items would have been better retained both on L-DOPA and on placebo, and that this effect would have been increased on L-DOPA. However, the reward manipulation did not work, and instead high and low reward pairs and locations were equally likely to be remembered both on L-DOPA and placebo.

See below for comparisons between low and high reward, and comparisons between L-DOPA and placebo.

		**Low reward** (mean ± SD)	**High Reward** (mean ± SD)	Wilcoxon test statistic	z	p-value
**Locations**	L-DOPA	0.631 (0.13)	.601 (0.2)	177	0.88	0.385
	Placebo	0.584 (0.19)	0.612 (0.16)	150	1.21	0.230
**Pairs**	L-DOPA	0.801 (0.15)	0.778 (0.18)	316.5	1.342	0.181
	Placebo	0.793 (0.17)	0.799 (0.18)	206	0.07	0.954

n = 34, across all tests. Accuracy reported in fractions. Therefore, overall maximum possible score would have been 1, and chance-level performance would have been ~.33.

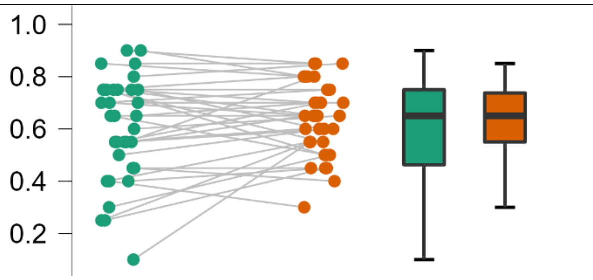

High (green) versus low (orange) reward accuracy for locations on L-DOPA

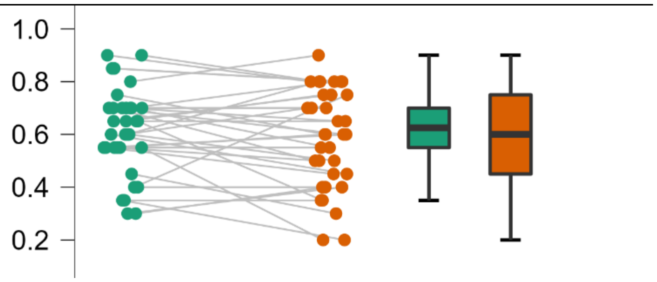

High (green) versus low (orange) reward accuracy for locations on placebo

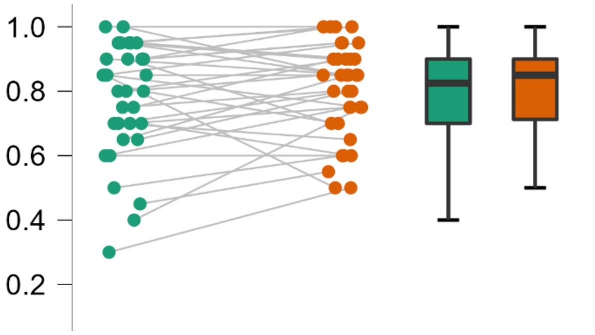

High (green) versus low (orange) reward accuracy for pairs on L-DOPA

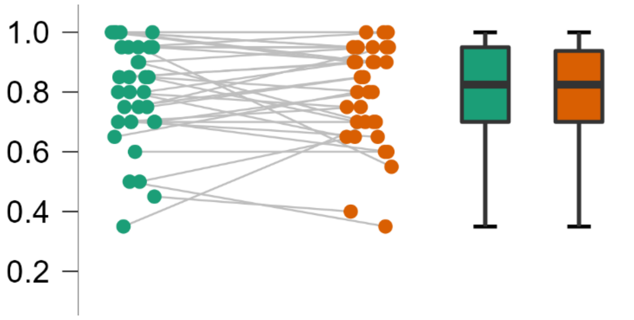

High (green) versus low (orange) reward accuracy for pairs on placebo

Supplementary Figure

		**L-DOPA** (mean ± SD)	**Placebo** (mean ± SD)	Wilcoxon test statistic	z	p-value
**Locations**	Overall	24.7 (6.2)	23.9 (6.4)	273	0.83	0.409
	High Reward	12.0 (4.0)	12.2 (3.2)	244.5	0.37	0.721
	Low Reward	12.6 (2.8)	11.7 (3.7)	263	1.37	0.127
**Pairs**	Overall	31.6 (6.0)	31.8 (6.3)	265	0.02	0.993
	High Reward	15.6 (3.6)	16.0 (3.5)	261.5	0.34	0.738
	Low Reward	16.0 (2.9)	15.9 (3.4)	248.5	0.44	0.665

n = 34, across all tests. There were 40 pairs, of which half were high and half were low reward. Therefore, overall maximum possible score would have been 40, and chance-level performance would have been ~13.33. Maximum score for Low / High reward items was 20, with chance level performance being ~6.66

6 SM6 : Study 2 design and results

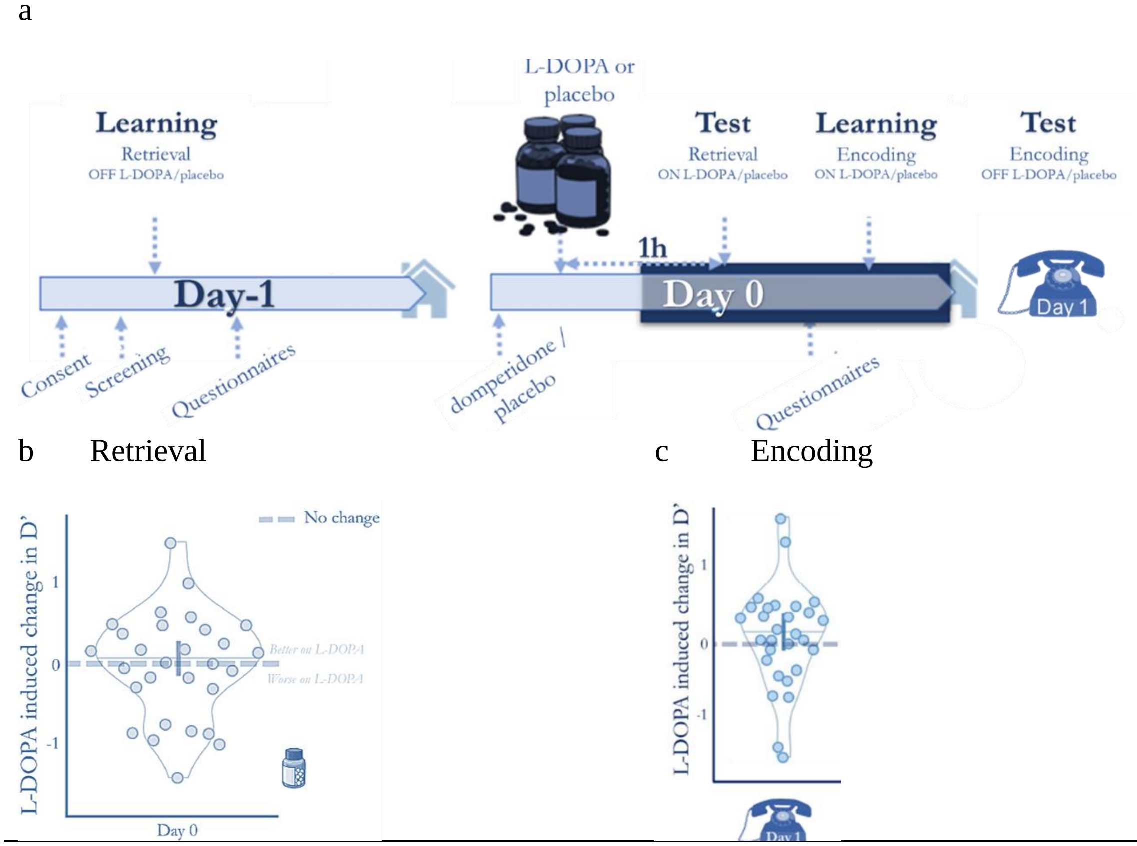

Supplementary Figure:

a) Study 2 timeline**:** Volunteers completed two identical test sets, each consisting of three days. On Day -1 (minus one) of each session the volunteers learnt a list of words. On Day 0 volunteers were dosed with L-DOPA or placebo before retrieval was tested (retrieval condition; L-DOPA active at the time of retrieval). After this they learnt another list of words. Their recall on this task was tested over the phone the following day (Day 1, encoding condition; L-DOPA active at the time of encoding). The shaded area show time L-DOPA is active in the body based on pharmacological profile. Study 2 aimed to test the effect of L-DOPA on retrieval and encoding. Questionnaires refer to demographic questionnaires reported in the demographics table SM2. This study was double-blinded, with dosing-order randomised and counterbalanced.

b) L-DOPA had no overall effect on memory test performance when active at retrieval. The individual dots denote performance change per participant (L-DOPA D' minus placebo D'). The dotted line denotes no difference. Dots above that line were participants whose performance was better on L-DOPA and below participants who performed worse on L-DOPA.

c) L-DOPA had no overall effect on encoding when memory was tasted 1 day after learning.

7 SM7 : Paired differences for delayed memory tests

Single exposure lists

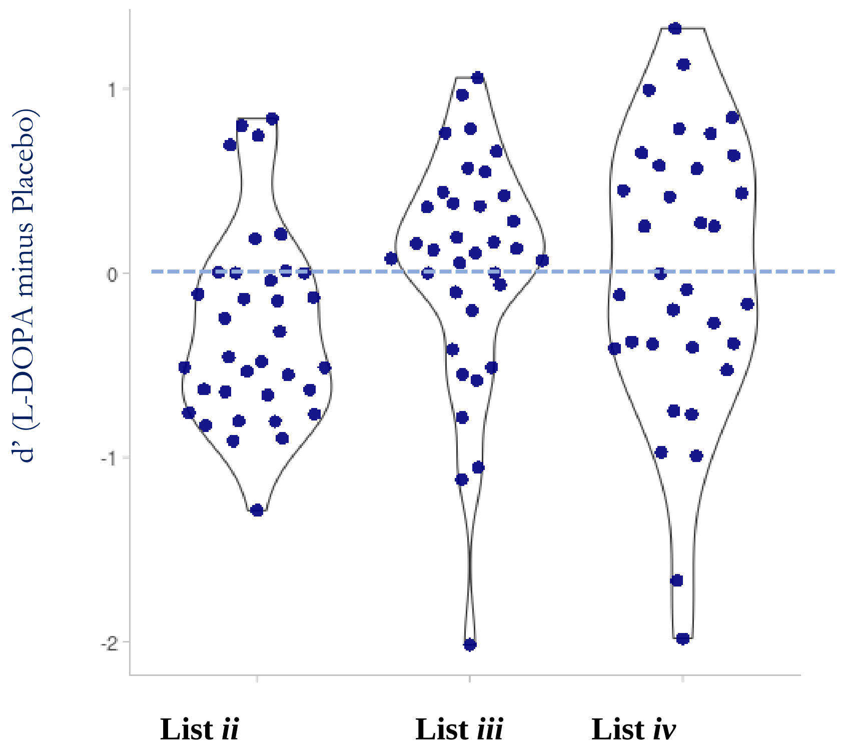

Supplementary Figure: Blue line denotes no difference. Data points above the blue line performed better following L-DOPA compared to placebo. This figure complements **Fig2a** in the main paper. **Fig2a** maps the individual performance for each participant for L-DOPA and placebo nights separately. Here, you can see the paired difference in performance.

Descriptive Statistics

	D1 (List ii)	D3 (List iii)	D5 (List iv)
**Mean difference (L-DOPA minus placebo)**	-0.294	0.037	-0.003
**Std. Deviation**	0.523	0.629	0.762
**Minimum**	-1.288	-2.017	-1.982
**Maximum**	0.839	1.060	1.327

8 SM8 : Original paired data for Manuscript figures 2, 3, and 4

a) **Figure 2c: D’ for re-exposed List *i* (left) and List *ii* (right) items on Day 1 for L-DOPA (green) and placebo (orange)**

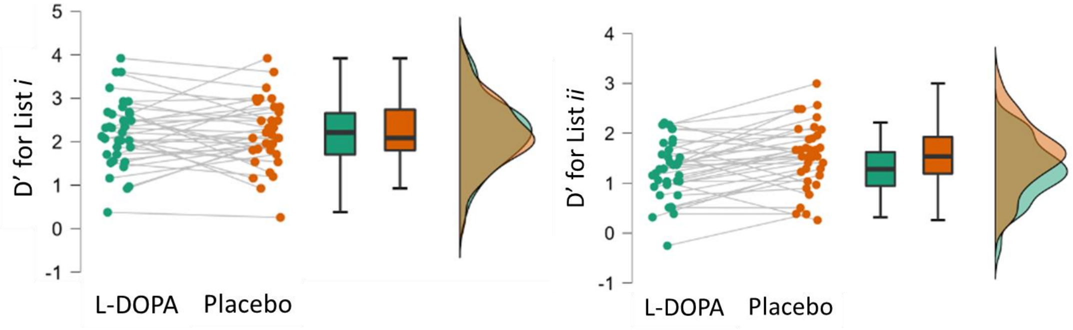

b) **Figure 3: Slow wave sleep durations for L-DOPA and (left) and placebo (right)**

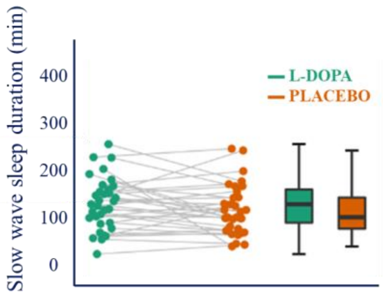

c) **Figure 4: Each of slow (0.6-1Hz), fast (1-4Hz) and SO (0.6-1) proportion of overall activity 0.6-4Hz activity**

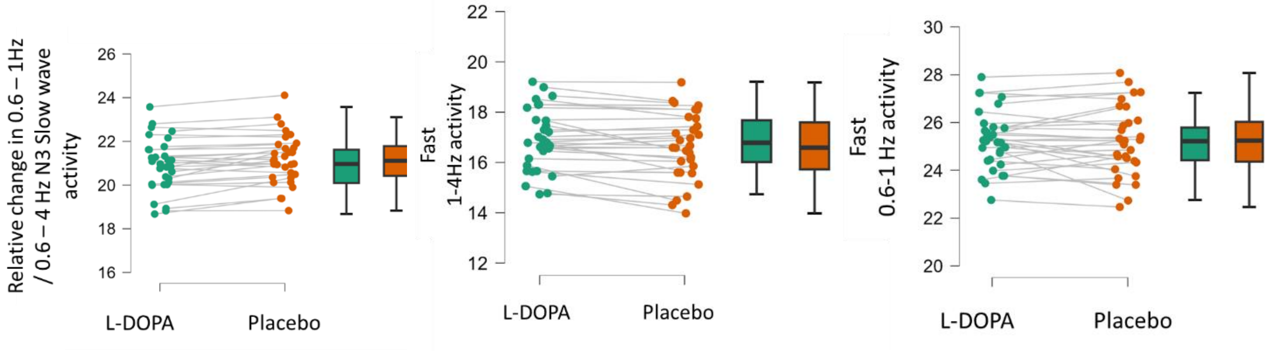

Supplementary Figure: Raw data plots to complement core text.

9 SM9 : Missing data

	Test day			
	**LDOPA**		**Placebo**	
	🏠 **0**	📞**1**	🏠 **0**	📞**1**
**# of volunteers**				
**1**	**X**	**X**		
**3**		**X**		
**1**		**X**	**X**	
**1**				**X**
**2**			**X**	**X**

Summary of missing data.
The above table provides a summary of missing data for the **SECONDARY** study. We recruited 35 volunteers to take part in the this control experiment. Two were excluded prior to dosing; due to a contraindication, and for participating in another drug trial simultaneously.
Crosses (X) denote missing data points for each test session with the left column denoting the number of volunteers affected. Remaining data for each volunteer was used except where the volunteer only completed one test session (n = 3, top and bottom rows,); two withdrew consent for personal reasons and one experienced significant nausea and vomiting as a side effect on their second testing session. Remaining data for 5 volunteers was missing partially, either due to missed phone calls or experimenter error, and one volunteer was excluded entirely as their accuracy being so low that it was below chance level – suggesting they had misunderstood the task – or due to experimenter error where wrong test versions were used.

Main study
Fifty-eight volunteers completed screening for the main experiment. Ten screened volunteers could not take part due to diary clashes (trial finished before they could be booked in), 2 could not take part due to incidental findings that were also contraindications revealed at screening, 3 could not take part due to existing cardiac or medicinal contraindications and a further 4 had other contraindications. 4 participants refused participation following screening (final n = 35). Further, data for one follow-up phone call (placebo, 📞Day 5) had to be excluded due to researcher error (same set of distractors and targets were used as on 📞Day 3). Data were partially or entirely missing for the polysomnography (PSG) for four volunteers due to technical errors at recording (n_{PSGSWS} = 31). One additional datapoint was excluded from spindle analyses as spindle data could not be reliably extracted ($n_{PSGSPINDLES}$ = 30)

10 SM10 : Re-exposed items were better retained following day across both conditions

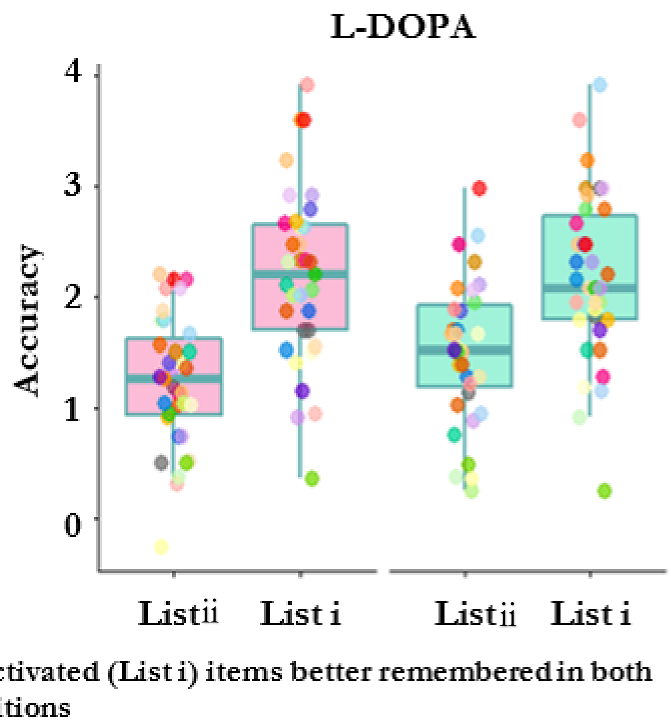

Supplementary Figure: Performance for re-exposed compared to other words (List i to List ii respectively) was better both ON and OFF L-DOPA, suggesting stronger memory traces due to re-exposure.

On both the L-DOPA ($t(34) = -8.419$, $p<.001$, $BF_{10} = 14300000$, error <.001%) and on placebo ($t(34) = -6.764$, $p<.001$, $BF_{10} = 165589$, error <.001%) re-exposed items ($mean_{L-DOPA} = 2.203 \pm 0.78$, $mean_{placebo} = 2.187 \pm .77$) were better retained on Day 1 than items that were not re-re-exposed ($mean_{L-DOPA} = 1.249 \pm 0.59$, $mean_{placebo} = 1.544 \pm .11$).

Boxplot lines show median and quartiles. Individual datapoints plotted

11 SM11 : List *i* accuracy on Day 0 and Day 1

a) Baseline performance on Day 0 **b) Memory performance on Day 1**

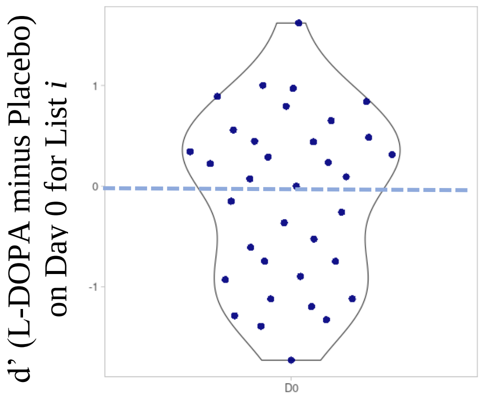

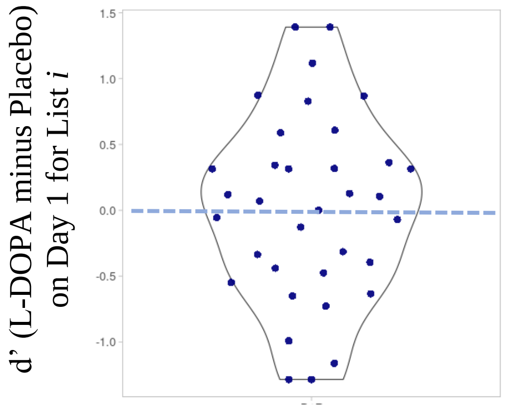

c) Performance change from day 0 to day 1 (day 1 minus day 0)

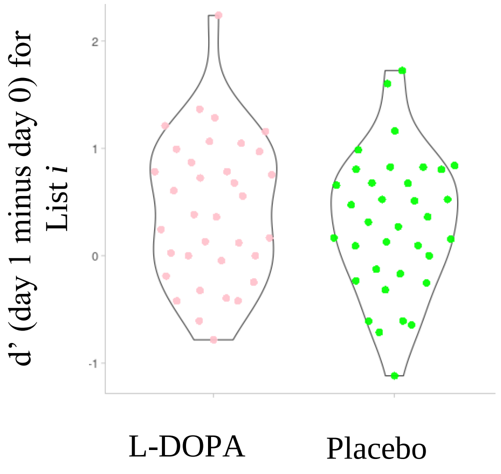

	Re-exposure	Day 1 test	Change on L-DOPA	Change on placebo
**Mean**	-0.118	0.016	0.432	0.298
**Median**	0.072	0.070	0.384	0.315
**Std. Deviation**	0.839	0.704	0.671	0.644

Supplementary Figure: All of these analyses are exploratory. The table shows summary statistics corresponding to data in figures. All n = 35

a. there was no difference in performance on baseline, t(34) = 0.831, p = 0.412, BF10 = 0.25, BF01 = 4.0, error <0.001
b. there was no difference in performance on day 1, t(34) = 0.134, p = 0.894, BF10 = 0.18, BF01 = 5.47, error <0.001
c. there was no difference in change in performance, t (34) = 0.906, p = 0.371, BF01 = 3.7, BF10 = 0.265, error <0.001

12 SM12 : L-DOPA disparately affects forgetting rate depending on memory strength

A

Variation source	Sum of squares	Mean square	Mean diff (std error)	Cohen's δ	F	p	BF_{01}	H_0 vs H_1
List type (*i* cf. *ii*)	22.293	22.293	- .798 (.08)	1.611	90.83	< .001	< . 001	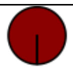
error	*8.345*	*.245*						
Treatment	.679	.679	- .139 (.08)	.280	2.75	.107	1.4	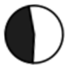
error	*8.406*	*.247*						
Interaction	.843	.843			6.15	.027	.7	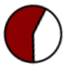
error	*4.658*	*.137*						

B

D' ~ List Type * Dose + (List Type + Dose || participant)

	Estimate (std error)	t	df	p	R^2 marginal
Intercept	1.800 (.10)	18.538	34.0	<.001	
List Type	.798 (.08)	9.460	34.0	<.001	
Dose	- .052 (.03)	- 1.882	29.0	.070	.261
List Type *Dose	.098 (.04)	2.302	32.8	.028	

L-DOPA has disparate effects on forgetting rate depending on whether items were reactivated or not.
A. Exploratory Parametric and Bayesian ANOVAs show a main effect of type of encoding (List i vs List ii) and an interaction between treatment (placebo vs L-DOPA) and encoding type. BF_{01} represents likelihoods of collecting our data under models that do not include the given source for variation (H_0 in white) compared to models that include the variation source (H_1 in dark). Note that BF_{01} which denotes how much likelier our data are under the null are reported as opposed to BF_{10} for easier interpretation. P-values are corrected for FDR using Benjamini-Hochberg procedure and accounting for 3 tests. All df = 34, 1.
B. A planned Mixed linear model showed that dose and encoding type together explain 26.1% of the variation in d'. As the ANOVA cannot account for dose-dependent effects, we conducted a mixed linear effects analysis with dose (mg/kg) and re-exposure (List i vs ii), and the interaction between the two, as fixed effects, and individual subjects as random effects (with slopes and intercepts). The model revealed a main effect of encoding type ($t(34) = 9.460$, $p < .001$) and a treatment * encoding type interaction ($t(32.8) = 2.302$, $p = .028$) but no evidence for a main effect of dose ($t(29) = -1.882$, $p = .070$). The model without the random effect predicted 26.1% of the variability in d'. In other words, L-DOPA enhanced performance selectively for re-activated items, while reducing performance for activated items. Note that estimates are mean-centred.

13 SM13 : Self-reported sleep measures

	Mean (SD) L-DOPA	Mean (SD) Placebo	Credible interval δ	t (df = 31)	p	BF_{01}	H_0 vs H_1
St Mary's Hospital Sleep Questionnaire							
Efficiency (%)	75.8 (15.6)	77.0 (22.9)	[- .378 – .275]	163*	*.156*	5.1	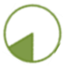
Onset latency (min)	17.1 (20.7)	27.4 (31.6)	[- .641 – .045]	61.0*	*.060*	1.2	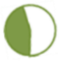
Latency (min)	390.5 (84.2)	384.5 (95.1)	[- .442 – .223]	186*	*.952*	4.3	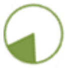
Maintenance score	3.1 (1.3)	2.8 (1.2)	[- .117 – .636]	174*	*.270*	2.4	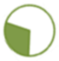
Sleep satisfaction**	62.1 (19.4)	67.5 (15.4)	[- .468 – .229]	- .694	*.494*	4.1	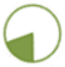
Wakefulness	31.8 (38.7)	27.8 (40.7)	[- .282 – .378]	.302	*.765*	5.1	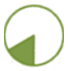
Leeds Sleep Evaluation Questionnaire				df = 34			
Total	4.2 (.86)	4.3 (.96)	[- .432 – .221]	- .668	*.509*	4.5	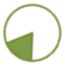
GTS	3.9 (1.7)	3.9 (.1.2)	[- .432 – .221]	.109	*.914*	5.5	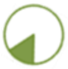
QOS	3.5 (1.8)	3.7 (1.8)	[- .432 – .221]	- .677	*.503*	4.5	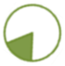
AFS	4.8 (1.6)	4.9 (4.9)	[- .432 – .221]	304.5	*.911*	4.9	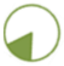
BFW	4.8 (1.6)	4.9 (1.4)	[- .432 – .221]	282.5	*.600*	4.8	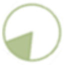

Sleep: Self-evaluation

We found no differences in self-reported measures of sleep quality. δ denotes effect size for the paired differences derived from the Bayesian posterior distribution. BF_{01} and H_0 vs H_1 show the probability of our data having been observed under the null (white) as opposed to the alternative (blue) hypothesis. All p-values are uncorrected. All errors <.06.
GTS = getting to sleep, QOS = quality of sleep, AFS = awakening from sleep, BFW =behaviour following waking.
*Wilcoxon's test; ** df = 28

Behavioural data: Data was entirely missing for the St Mary's Hospital Sleep Questionnaire (SMHSQ) for two volunteers following the L-DOPA and one volunteer following the placebo night (n_{SMHSQ} = 32). One volunteer on placebo, and two on L-DOPA, had omitted answers on the SMHSQ, so their sleep satisfaction score (SSS) could not be determined (n_{SSS}=29). These questionnaires were otherwise scored. The wakefulness after sleep onset score was calculated as the difference between self-reported sleep onset time and final wake up time, and self-reported sleep latency. Some volunteers reported less time between sleep onset and waking than spent asleep. For these nights, wakefulness after sleep onset was changed to 0 minutes to avoid negative values. There was no missing data for the Leeds Sleep Evaluation Questionnaire.

14 SM14 : L-DOPA increases spindle amplitude

a

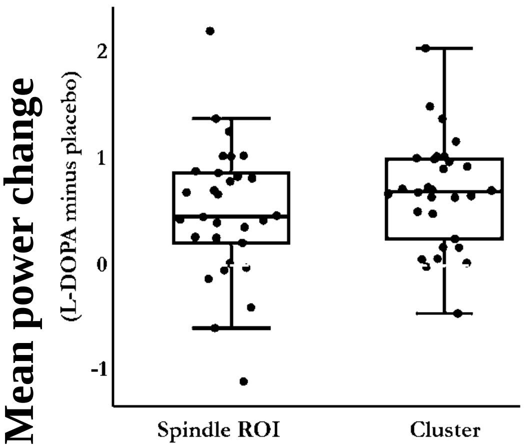

b

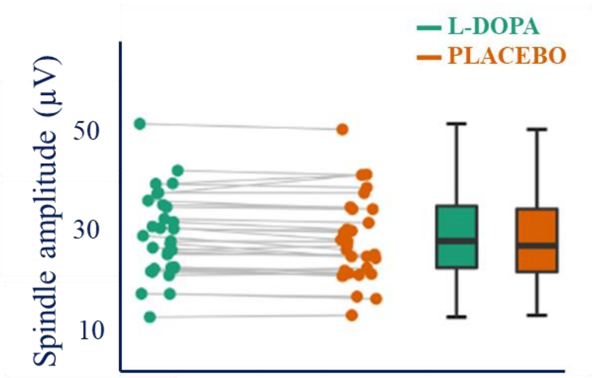

Supplementary Figure:

a) Individual mean power change between L-DOPA and placebo conditions during slow oscillation - spindle co-occurrence events, with respect to the 2 time-frequency areas of interest: the a-priori selected spindle region of interest, 11 - 16Hz, -0.5 - 0.5s centered on the slow oscillation peak; and the primary cluster revealed by the cluster permutation method**.** The power change for each individual was calculated by taking a mean for all power difference data points within these time-frequency spaces. While both areas were consistent across subjects, there is a very high level of consistency shown for the L-DOPA induced power increase.

b) Mean spindle amplitude during slow wave sleep for L-DOPA (left) and Placebo. This figure compliments Figure 5a in core text.

15 SM15 : Correlations between polysomnography measures and memory

	SWS duration and D’ on Day 1	
**N = 33**	List i	List ii
**Correlations on L-DOPA**		
Spearman’s rho	0.043	0.065
p-value	*0.810*	*0.720*
**Correlations on placebo**		
Spearman’s rho	0.450	0.320
p-value	*0.009*	*0.071*
	*P corrected = 0.012 **	

**Benjamini Hochberg corrected p-value accounting for each analysis in this table*

	SWS Spindle amplitude and D’ on Day 1	
**N = 31**	List i	List ii
**Pearson Correlations on L-DOPA**		
Pearson’s R	-0.026	-0.059
p-value	*0.891*	*0.753*
**Pearson Correlations on placebo**		
Pearson’s R	0.234	0.264
p-value	*0.151*	*0.204*

16 SM16 : Study 2 Results

Encoding

	Mean (SD) L-DOPA	Placebo	Credible interval δ	t	p	BF_{01}	H_0 vs H_1
**Day 1**				df = 28			
D’	1.257 (.76)	1.149 (.61)	[- .295 – .426]	- .352	.728	4.6	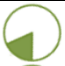
Criterion	- .027 (.35)	- .083 (.39)	[- .310 – .414]	114*	.197	4.7	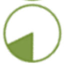
**Day 3**				df = 25			
D’	.865 (.47)	.710 (.50))	[- .001 – .763]	-2.128	.043	.7**	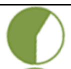
Criterion	- .107 (.40)	- .126 (.46)	[- .352 – .368]	- .040	.968	4.8	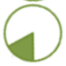
**Day 5**				df = 26			
D’	.692 (.45)	.592 (.40)	[- .317 – .588]	- .325	.748	2.4	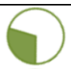
Criterion	- .147 (.38)	- .167 (.33)	[- .334 – .378]	- .148	.884	4.9	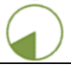

2nd clinical trial Encoding: Pairwise comparisons

* Wilcoxon test used due to non-parametric data. All samples contained zero values for paired differences. P-values for Wilcoxon tests for such data are less reliable

Only Day 1 results are reported the manuscript. However, memory was also prompted on days 3 and 5 as described in SM5. The trend towards L-DOPA induced improvement in d’ at day 3 does not survive multiplicity corrections and therefore is not interpreted further in this manuscript.

**Retrieval**	Mean (SD) Day preceding L-DOPA	Placebo	Credible interval δ	t	p	BF_{01}	H_0 vs H_1
Day -1	L-DOPA	Placebo		df = 27			
D’	2.842 (.72)	2.721 (.76)	[- .180 – .538]	-1.041	.307	3.1	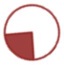
Criterion	.114 (.34)	.196 (.35)	[- .564 – .149]	-1.198	.241	2.6	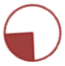
Day 0	L-DOPA	Placebo		df = 27			
D’	1.658 (.55)	1.609 (.56)	[- .278 – .417]	.393	.698	4.6	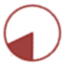
Criterion	- .216 (.39)	- .192 (.47)	[- .384 – .316]	- .224	.968	4.9	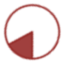

Before L-DOPA / Placebo (Day -1)

2nd study: Retrieval: Pairwise comparisons
